# Decoding the unseen: unsupervised anomaly detection in metal–organic frameworks for discovery beyond the norm

**DOI:** 10.1039/d5sc06431g

**Published:** 2026-02-24

**Authors:** Hosein Alimardani, Shayan Abaei, Mehrdad Asgari

**Affiliations:** a Faculty of Engineering, University of Tehran Tehran Iran; b School of Chemical Engineering, Faculty of Engineering, University of Tehran Tehran Iran; c Lucy Cavendish College, University of Cambridge Cambridge CB3 0BU UK ma2000@cam.ac.uk

## Abstract

The discovery of chemically novel or structurally anomalous metal–organic frameworks (MOFs) is essential for expanding reticular design space and enhancing dataset reliability. We present CHEM-AD (Chemically Unusual Metal–organic Frameworks *via* Autoencoder-based Detection), a label-free, CPU-efficient pipeline that detects anomalous MOFs using 81 engineered descriptors (32 geometric/chemical/topological scalars plus a 49-dimensional metal-composition encoding). A compact symmetric autoencoder (∼1.8 × 10^5^ trainable parameters) learns the latent distribution of typical MOFs and assigns anomaly scores based on reconstruction error. Applied to 26 025 entries from MOFxDB, CHEM-AD identifies 488 outliers (∼1.87%) featuring distinctive topologies, unusual pore metrics (PLD: 2.56–29.48 Å; LCD: 4.89–63.59 Å), and extreme densities (0.057–4.27 g cm^−3^). These anomalies consistently occupy peripheral clusters in PCA embeddings and exhibit substantial Mahalanobis distances from normal MOFs, indicating multivariate deviation. Feature attribution reveals *connectivity* (*e.g.*, edge/node counts, degree dispersion) as the primary driver of anomalies, followed by window-limited geometry and linker–metal composition. We categorize results into three groups: (A) topologically unusual yet plausible candidates, (B) anomalies with chemically resolvable issues, and (C) likely structural artifacts. The full pipeline executes in under six minutes on standard CPUs and does not require 3D structure fitting or graph parsing. CHEM-AD generalizes to other porous materials, providing a scalable framework for discovery, database curation, and robust preprocessing in materials informatics.

## Abstract 2

**fo** Discover chemically novel MOFs using CHEM-AD, an unsupervised machine learning pipeline. We leverage autoencoders for structural anomaly detection to expand reticular design and enhance material dataset reliability.

## Graphical abstract

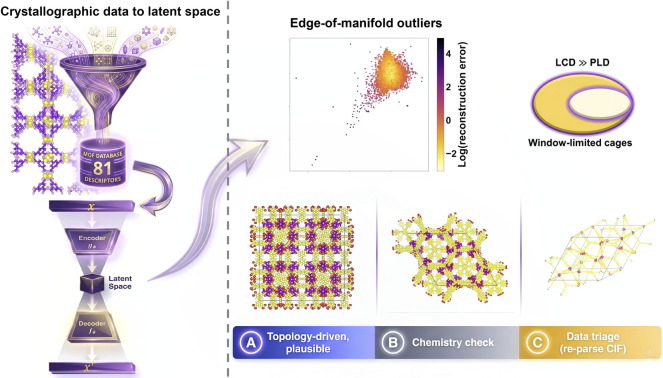

## 1. Introduction

Metal–organic frameworks (MOFs) represent one of the most structurally diverse and functionally tunable families of porous crystalline materials, formed by coordination of metal nodes with organic linkers into extended reticular architectures.^1,2^ Their ultrahigh surface areas, modular building blocks, and chemical flexibility have led to wide-ranging applications in gas storage,^3,4^ carbon capture,^5^ catalysis,^6^ chemical sensing,^7^ and drug delivery.^8^ The accelerating discovery of both experimental and hypothetical MOFs has yielded extensive databases such as CoRE MOF,^9^ the CSD MOF subset,^10^ and MOFxDB—enabling^11^ data-driven design and screening workflows.^12,13^

While this rapid expansion in structural repositories is a catalyst for materials discovery, it introduces a new and pressing challenge: how can we automatically identify chemically unusual, mislabeled, or genuinely novel MOFs that deviate from established design paradigms? Standard classification or regression models—whether descriptor-based^14–16^ or graph neural network (GNN)-based^17,18^—typically smooth over structural outliers or treat them as noise, missing opportunities to uncover novel topologies, experimental artifacts, or data inconsistencies. However, these anomalies may indicate new opportunities for material discovery or signal critical database curation needs.^12,19^

Recent work has also shown that “computation-ready” MOF databases themselves contain a surprisingly high fraction of chemically invalid structures. White and colleagues recently introduced MOSAEC, an automated validator that checks metal oxidation states and related chemical heuristics, and reported structural error rates exceeding 40% across several widely used experimental and hypothetical MOF databases, including CoRE MOF.^20^ This and related efforts underscore that two complementary problems must be addressed in data-driven MOF discovery: detecting outright structural errors (*e.g.* impossible oxidation states or charge imbalance), and surfacing structurally unusual yet chemically plausible frameworks that sit at the edge of known design space. CHEM-AD is designed to tackle the latter, while its sanity flags provide a lightweight, oxidation-state-aware triage layer that can be used alongside more specialized validators such as MOSAEC.

In this context, an “anomalous MOF” is not a mysterious black-box label but a structure that sits at the edge of the learned distribution of known frameworks. Specifically, such anomalies fall into a few physically interpretable categories. Some are *ultra-porous outliers*, with extremely low densities and very large pores that are unlikely to be mechanically stable and often indicate database or parsing artefacts. Others are *window-limited frameworks*, where very large internal cavities are connected by relatively narrow windows; these materials can exhibit unusual diffusion and adsorption behaviour and are rare in existing datasets. A third group consists of *topologically unusual but chemically plausible* MOFs, whose pore sizes and densities look ordinary but whose connectivity patterns (ring tilings, node degrees, and interpenetration) deviate strongly from common nets such as *pcu*, *fcu*, or *soc*. In all three cases, high anomaly scores correspond to structures that are either scientifically interesting or worth flagging for curation, rather than to arbitrary numerical outliers.

Furthermore, the vastness and heterogeneity of current MOF datasets make manual inspection impractical and supervised learning unreliable in the absence of ground-truth labels or comprehensive structural typologies. An unsupervised, scalable, and interpretable approach is therefore essential to uncover hidden signals of novelty and error within these high-dimensional chemical landscapes.

Recent advances in unsupervised learning methods, such as autoencoders,^21^ variational encoders,^22^ and contrastive learning^23,24^ have demonstrated a strong potential in detecting anomalies and learning chemically meaningful embeddings without labeled data. Dimensionality reduction tools such as principal component analysis (PCA) and t-distributed stochastic neighbor embedding (t-SNE) further enhance the interpretability of complex feature spaces, enabling researchers to visualize and isolate distinct structural motifs or outlier clusters.

In this work, we introduce CHEM-AD (Chemically Unusual Metal–organic Frameworks *via* Autoencoder-based Detection), a data-efficient, unsupervised anomaly detection pipeline tailored to the structural diversity of MOFs. Operating on a standardized set of geometric, topological, and chemical descriptors, CHEM-AD leverages a compact autoencoder architecture to learn the manifold of typical structures and flag anomalous entries based on reconstruction error—without relying on topological labels or 3D model parsing. Unlike conventional supervised or clustering techniques that often overlook rare or mislabeled structures, CHEM-AD prioritizes such deviations. This approach offers a principled route to identify chemically meaningful outliers, guide database curation efforts, and improve the integrity of training sets for downstream machine learning workflows. While demonstrated on over 26 000 entries from MOFxDB, the framework generalizes to other porous materials such as COFs, zeolites, and porous polymers, providing a scalable tool for large-scale screening and longitudinal database monitoring.

Throughout this work, we use the term “meaningful anomaly score” to describe exactly this situation: high scores that consistently correspond to physically interpretable deviations in porosity and topology (*e.g.* ultra-low density, window-limited cavities, or rare connectivities), rather than to noise, single-feature outliers, or numerical instabilities. We show below that CHEM-AD concentrates its highest scores on these edge-of-manifold frameworks and that the corresponding structures can be explained in terms of their descriptor patterns and unit-cell geometries.

## 2. Methods

This section outlines the full pipeline of the CHEM-AD framework, including data preprocessing, descriptor normalization, unsupervised model architecture, anomaly scoring, dimensionality reduction, visualization, feature attribution, and validation. The goal is to systematically identify chemically and structurally unusual MOFs within the MOFxDB dataset using only tabular descriptors in an unsupervised and CPU-efficient manner.

### 2.1. Dataset: MOFxDB descriptor matrix

We employed the MOFxDB dataset,^11^ a curated, computation-ready collection of over 160 000 structurally and chemically diverse metal–organic frameworks that aggregates adsorption-ready structures from multiple sources, including both experimentally reported and hypothetical MOFs (*e.g.* CoRE MOF 2019 and topologically enumerated hMOF libraries). To balance computational efficiency with chemical coverage, we did not train on the full ∼160 000-entry matrix. Instead, we constructed a mixed but controlled subset consisting of (i) all CoRE MOF 2019 structures present in MOFxDB (experimentally reported, solvent-free CSD MOFs) and (ii) a random subset of 15 000 MOFs from the MOFxDB “edge” category. We did not perform an additional deduplication step on the CoRE MOF subset used in this work. Instead, we relied on the upstream curation of the CoRE MOF 2019 database, which includes an explicit duplicate-removal procedure (DOI-based filtering and CIF-to-CIF identity checks using lattice/volume/composition/atomic positions, cross-checked against StructureMatcher-style matching). Any residual near-duplicates (*e.g.*, closely related variants reported under different conditions) would primarily re-weight common motifs in an unsupervised setting rather than creating spurious anomalies. In the original MOFxDB work, this edge pool denotes frameworks that lie near the extremes of the descriptor distributions (*e.g.* very high surface area, very low or very high density, unusual compositions) and is therefore enriched in geometrically and chemically atypical structures. This design ensures coverage of both well-established and structurally extreme regions of MOF space while keeping the data volume tractable for repeated unsupervised training, cross-validation, and ablation studies. After combining these two subsets, rows with any missing descriptor values were removed, yielding a clean set of 26 025 unique MOFs. Unless otherwise stated, aggregate statistics (*e.g.* PCA embeddings and score distributions) are reported over this combined experimental–hypothetical subset. Each MOF entry is annotated with a comprehensive set of engineered descriptors derived from structural analyses, CIF parsing, and geometric computations.

The raw descriptor matrix consists of:

• Geometric features: framework volume, framework density, volume per atom, specific surface area (m^2^ g^−1^ and m^2^ cm^−3^), void fraction, pore limiting diameter (PLD), and largest cavity diameter (LCD). Core metrics such as PLD, LCD, and surface areas were obtained directly from the MOFxDB repository, while complementary volumetric measures and void fractions were derived from geometric analysis of the CIF structures.

• Chemical features: number of atoms, average atomic mass, average electronegativity, electronegativity variance, metal fraction, number of unique elements, metal atom count, linker atom fraction, mean and standard deviation of linker bond lengths, and mean metal coordination number. In a dedicated ablation study (SI, Section S.4), we further augment this descriptor block with an explicit 49-dimensional multi-hot metal-composition vector that encodes the presence or absence of each metal element (Sc–Lu). All chemical descriptors were computed directly from the CIF files using robust structural and compositional analysis tools implemented in pymatgen.^25^

• Topological features: average node connectivity, average ring size, mean coordination number, degree assortativity, mean degree centrality, graph density, graph entropy, graph transitivity, largest connected component fraction, node connectivity standard deviation, number of connected components, number of edges, and number of nodes. These descriptors were obtained by transforming CIF-derived frameworks into periodic graphs and applying network-theoretic analysis to capture connectivity and symmetry attributes.

For all descriptors that are not directly provided by MOFxDB, CIF files were processed with in-house Python workflows. Core geometric quantities such as PLD, LCD, geometric surface areas, density and void fraction are taken directly from the MOFxDB tables, which in turn are computed with Zeo++ as described.^11^ Additional volumetric measures (*e.g.* framework volume and volume per atom), as well as all chemical descriptors (atomic counts, mean atomic mass, electronegativity statistics, metal fractions, numbers of unique elements, linker bond-length statistics, and mean metal coordination numbers), were computed from the CIF files using pymatgen.^25^ For topological descriptors, each MOF was converted into an undirected periodic graph by treating metal and linker nodes as vertices and placing edges between atoms within a distance cutoff consistent with the bonding information in MOFxDB. The resulting graphs were then analysed with standard network-analysis tools (*e.g.* networkx) to extract node and edge counts, degree statistics, assortativity, transitivity, graph density and entropy, largest-component fraction, node-connectivity dispersion, and related measures. This workflow ensures that geometric, chemical and graph-based descriptors are fully determined by the CIF input and MOFxDB tables and can be reproduced independently of the anomaly-detection model.

The resulting descriptor matrix comprised *d* = 81 numerical features per MOF, integrating quantities sourced directly from MOFxDB with those computed *via* pymatgen and graph-analysis workflows, ensuring comprehensive representation of structural, chemical, and topological diversity. Also for interpretability, the feature-attribution and correlation analyses in Section 3.6 and Fig. 8 show attributions of all descriptors and 9 focus on the 32 scalar geometric/chemical/topological descriptors; the 49-dimensional metal-composition vector ablation study is thoroughly discussed in SI Section S.4.

### 2.2. Preprocessing and normalization

Preprocessing was conducted by partitioning the features into numeric and one-hot subsets. Only the numeric features were standardized. Normalizing the one-hot features can create artificial relationships between categories. Moreover, the scaler was fitted exclusively on the training data to prevent data leakage. The transformation is defined as:
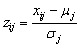
where *x*_*ij*_ is the value of descriptor *j* for sample *i*, and *µ*_*j*_, *σ*_*j*_ are the mean and standard deviation of descriptor *j* across all training samples. The final input tensor was formed by concatenating these standardized numeric features with the untouched one-hot encoded features. This pipeline was implemented using scikit-learn's StandardScaler.

The processed matrix *X* ∈ ℝ^*n*×*d*^, where *n* is the number of MOFs, served as the input to the autoencoder.

### 2.3. Autoencoder design and training

A fully connected, symmetric autoencoder was implemented to project the 81-dimensional MOF descriptor space into a compact latent representation and subsequently reconstruct it. The architecture was designed to capture dominant structural–chemical patterns in an unsupervised manner, without prior class definitions.

The encoder maps each input descriptor vector *x* ∈ ℝ^81^ (one MOF) to a 16-dimensional latent vector *z*, and the decoder reconstructs an output *x̂* ∈ ℝ^*d* = 81^ from *z*. The full autoencoder contains on the order of 1.8 × 10^5^ trainable parameters (weights and biases across all layers). The reconstruction error for a dataset of *n* MOFs is quantified by the mean-squared error (MSE), averaged over all samples and features:
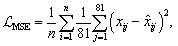
where *x*_*ij*_ is the *j*-th feature of the *i*-th input vector and *x̂*_*ij*_ is the corresponding reconstructed feature.

Since the dataset is unlabeled, the reconstruction MSE alone is an insufficient metric for model selection. A model that achieves a low average MSE can effectively behave as a “lazy generalist”, capable of reconstructing all inputs—including anomalies—with high fidelity. This behaviour is counterproductive, as the primary goal is not perfect reconstruction but to learn a representation that *exaggerates* the difference between normal and anomalous data. To this end, we employ a composite evaluation approach that targets latent-space separability.

For a given trained autoencoder, we first calculate the reconstruction MSE for every sample in the training set. We then apply the elbow method^26^ to the sorted list of these error scores to obtain an objective, data-driven anomaly threshold. This threshold is subsequently applied to the test set to generate provisional labels (“normal” or “anomalous”).

The latent vectors of the normal and anomalous test samples are then projected into a principal component analysis (PCA) space preserving 95% of the total variance, thereby reducing noise and ensuring a well-conditioned covariance matrix for reliable distance estimation. Within this reduced and decorrelated subspace, the Mahalanobis distance—computed using the centroid and covariance of the normal data—captures how strongly anomalous embeddings deviate from the statistical distribution of normal representations, providing a distribution-aware criterion that emphasises latent-space separability over reconstruction fidelity.

Within this PCA space, we define a Mahalanobis distance ratio (MDR) as
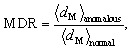
where 〈*d*_M_〉_anomalous_ and 〈*d*_M_〉_normal_ denote the mean Mahalanobis distances of the anomalous and normal test samples, respectively. Higher MDR values indicate better separation between normal and anomalous MOFs in latent space.

To systematically identify an effective architecture, a grid search was performed over latent dimensionalities and hidden-layer compression rates. The latent dimension was varied as *d*_latent_ ∈ {2, 4, 8, 16, 32, 64}, while the hidden-layer configuration was governed by a tapering-size parameter *t* ∈ {1, 2, 4, 8, 16, 32, 64}. The tapering size specifies the number of neurons reduced between consecutive layers, progressively compressing the input dimension (81) toward the latent bottleneck. For instance, a tapering size of *t* = 1 results in a gradual compression (81, 80, 79, …, latent), whereas larger tapering sizes produce shallower networks with more aggressive dimensionality reduction between layers.

Each (*d*_latent_, *t*) configuration was evaluated across five randomised train–validation–test splits (60%/20%/20%) to ensure robustness and generalisability. For each split the autoencoder was trained for 200 epochs, and the MDR was computed exclusively on the test set, so that architecture selection relied solely on unseen data. Architectures achieving a high MDR with low variability across the five splits were considered more reliable, as they consistently produced latent spaces that effectively separated normal and anomalous data distributions. A detailed account of this architectural optimisation, including full MDR statistics, is provided in the SI (Section S3).

Training protocol.

• Optimizer: Adam (learning rate = 1 × 10^−3^).

• Regularization: L2 weight decay with *λ* = 0.001.

• Normalization: batch normalization after each hidden layer.

• Loss function: reconstruction MSE, monitored on the validation set.

• Epochs: 200 per train–validation–test split.

• Batch size: 32.

• Data split: 60% training, 20% validation, 20% test, repeated over five random splits.

• Callbacks:

– *EarlyStopping*: monitored on validation loss with patience = 50 and restoration of best weights.

– *ReduceLROnPlateau*: monitored on validation loss, factor = 0.2, patience = 25, minimum learning rate = 1 × 10^−5^

• Shuffling: enabled at each epoch.

• Framework: TensorFlow 2.13 (CPU-only).

Network architecture.

• Input layer: 81 neurons (descriptor dimension).

• Encoder: sequence of fully connected hidden layers starting from 81 neurons and decreasing by 2 neurons per layer (tapering size *t* = 2) down to 17 neurons.

• Latent (bottleneck) layer: 16 neurons, ReLU activation.

• Decoder: mirror of the encoder, with hidden layers increasing from 17 back to 79 neurons (tapering size *t* = 2).

• Output layer: 81 neurons, linear activation.

Rows with missing descriptor values (NaNs) were removed prior to model fitting, yielding a clean dataset of 81 features per MOF. Training converged within approximately 6 minutes on a standard 8-core Intel i7 CPU, reaching mean training, validation, and test MSEs of 0.3204, 0.3954, and 0.3588, respectively, across the five random splits. This indicates robust reconstruction performance without overfitting. Further details of the architecture design, optimisation strategy, and PCA profile are provided in the SI (Section S3). For the final anomaly analysis, we keep a strict separation between the data used to *define* the detector and the data used to *evaluate* it. The autoencoder weights, the feature scalers, and the reconstruction-error threshold are all fitted using only the training portion of each split. Using the training reconstruction errors to calibrate the threshold is not a form of data leakage in this unsupervised setting; rather, it defines what the model considers to be “normal” behaviour. Once the model and threshold are fixed, anomaly scores for any MOF—whether it belongs to the training, validation, or test subset, or to a new structure outside MOFxDB—are obtained by applying the same pre-processing, passing the descriptor vector through the trained autoencoder, and computing its reconstruction MSE. The anomaly decision then simply compares this score to the training-derived threshold. Finally, although we fix the 81-dimensional descriptor set in this study, the implementation is fully modular: users may supply alternative feature matrices (*e.g.* purely topological or purely geometric descriptors) and retrain CHEM-AD using the same MDR-based hyperparameter search to obtain specialised anomaly detectors tailored to specific descriptor families.

### 2.4. Anomaly score calculation

After training, the model was used to reconstruct all input vectors. An anomaly score was computed for each MOF as the mean squared reconstruction error:
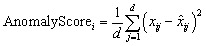


MOFs with high anomaly scores deviate significantly from the learned descriptor manifold and are flagged as potential structural or chemical outliers.

Once trained on this 26 025-entry subset, CHEM-AD can be applied in a purely feed-forward manner to assign anomaly scores to any additional MOFs that are represented in the same descriptor space. Scoring the remaining MOFxDB entries therefore requires only a single pass through the frozen scalers and autoencoder, with cost linear in the number of structures. In practice, this means the present model can be reused to screen the full MOFxDB pool or new hypothetical libraries without retraining.

### 2.5. Dimensionality reduction and visualization

To qualitatively assess the anomaly structure and visualize descriptor-space embeddings, we applied PCA,^27^ t-distributed Stochastic Neighbor Embedding (t-SNE):^28^

• PCA: a deterministic method to obtain the global structure of the data.

• t-SNE: used to project *X* and the latent layer *Z* into 2D; parameters: perplexity = 75, iterations = 1000.

The resulting 2D embeddings were colored by anomaly score to visually differentiate between regular and anomalous MOFs. These visual tools offer a map of the descriptor landscape with high-anomaly regions easily identifiable.

### 2.6. Latent-space characterization and feature attribution

As described in Section 2.3, we determine an anomaly threshold *τ*_elbow_ by applying the elbow method to the sorted distribution of training-set reconstruction errors. Here we use this fixed *τ*_elbow_ to convert continuous scores into binary anomaly labels for evaluation and visualisation. This knee point provides an objective, reproducible operating threshold for defining the “anomalous” subset in our quantitative analyses. In practical use, however, a practitioner can also work down the ranked list and visually inspect structures in descending anomaly score until they no longer appear chemically or structurally unusual. Our detailed inspection of the top-ranked MOFs in Section 3.4 follows exactly this strategy and confirms that high scores correspond to chemically meaningful outliers.

Let *τ* be the elbow threshold and define the anomaly set 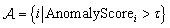 with size 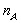.

Inputs *x*_*ij*_ (standardised descriptors) are reconstructed as *x̂*_*ij*_ by the autoencoder, where *i* indexes MOFs and *j* indexes features. For the feature-attribution analysis we consider *d* descriptors (here *d* = 32 numeric features; see Section 2.1). For sample *i* and feature *j* we define the absolute reconstruction error*e*_*ij*_ = |*x*_*ij*_ − *x̂*_*ij*_|.

Restricting to the anomalous set 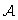 of size 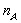, the mean error for feature *j* is
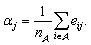


The vector ***α*** = (*α*_1_, …, *α*_*d*_) collects the per-feature contribution values visualised in Fig. 8. For visualisation we *L*_1_-normalise this vector to obtain shares that sum to one,
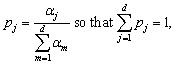
where *d* is the number of descriptors included in the attribution analysis. No contrasts to the normal set and no additional weighting are applied; the display mirrors the actual workflow: select all MOFs with AnomalyScore_*i*_ > *τ* and compute *α*_*j*_ by averaging *e*_*ij*_ over 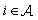.

### 2.7. Validation and triage protocol

We validated anomalies in two stages, a lightweight battery of rule-based checks followed by a distributional distance screen; so that high scores reflect chemically interpretable deviations rather than trivial artifacts. Let *τ* be the elbow threshold from the anomaly-score curve and define 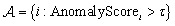 with 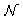 the remainder.

The ChemSanity flag ensures basic compositional and bonding plausibility (at least one metal atom; 1–10 unique elements; nonnegative electronegativity variance; linker bond-length mean within 0.9–2.2 Å and standard deviation within 0.0–0.4 Å). The GeoSanity flag enforces physically sensible porosity windows (PLD 1–50 Å, LCD 1–80 Å, void fraction in (0, 0.95), density 0.1–3.0 g cm^−3^, and surface area <10^4^ m^2^ g^−1^); PLD/LCD/SA were read from MOFxDB, whereas the remaining quantities were computed from the CIFs. TopoOK encodes a basic connectivity sanity check on the periodic graph. For each MOF we construct an undirected periodic network and compute the size of its largest connected component as a fraction of all framework nodes. If this largest component accounts for at least 90% of the nodes and the mean node degree lies within a reasonable window for MOFs (*e.g.* 3–8), we set TopoOK = pass; otherwise we set TopoOK = fail. TopoOK therefore flags entries that either fragment into several comparable components (*e.g.* disconnected nets) or exhibit implausibly low or high connectivity. Highly interpenetrated frameworks may be marked as TopoOK = fail if their periodic graph resolves into multiple similarly sized components, but in such cases the flag is interpreted as a marker of topological complexity rather than as evidence of chemical impossibility. We additionally flag TopoOK = fail when the periodic graph decomposes into multiple connected components (*i.e.* num_connected_components > 1), which commonly occurs for interpenetrated nets or for entries where bond perception fragments the framework.

Buildable is a pipeline-validity flag indicating whether an entry can be successfully instantiated and processed end-to-end by our analysis workflow without fatal errors. We set Buildable = pass if the CIF can be parsed into a consistent periodic structure and all required quantities for downstream screening (descriptor vector construction and subsequent analyses) are available and finite; otherwise we set Buildable = fail. We interpret Buildable failures primarily as data-integrity or preprocessing issues (*e.g.* incomplete records, parsing failures, or missing/invalid descriptor values), rather than as a direct statement about chemical plausibility.

In the second stage, we quantify how far each standardized descriptor vector *x*_*i*_ sits from the typical population by the Mahalanobis distance*M*_*i*_ = (*x*_*i*_ − *µ*)^T^*Σ*^−1^(*x*_*i*_ − *µ*),with (*µ*, *Σ*) estimated on the full standardized dataset. Comparing *M*_*i*_ against the autoencoder score helps separate structures that are merely noisy in a few descriptors from those that are genuinely distributional outliers in the multivariate sense.^29^ We emphasize that *M*_*i*_ is computed in the full standardized descriptor space and should not be interpreted as the Euclidean distance between points in the 2D PCA visualizations (which are lossy projections).

The Results report these diagnostics in three views: (i) density *vs.* surface area and PLD *vs.* LCD scatter plots with 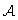 highlighted; (ii) autoencoder score *vs. M*_*i*_ with the threshold *τ* marked, alongside a bar chart of the top-10 anomaly scores; and (iii) a triage table listing ChemSanity, GeoSanity, TopoOK, and Buildable for the highest-scoring candidates.

These sanity flags are not a pre-existing standard or a dedicated software package; rather, they are deliberately simple, rule-based diagnostics introduced in this work. They summarise widely used physical and structural plausibility checks in MOF screening (*e.g.* reasonable density and porosity windows, connectivity and coordination constraints) that are commonly applied when curating experimental and hypothetical MOF databases.^1,30,31^ In particular, the GeoSanity thresholds are chosen to be deliberately conservative. For example, ultraporous MOFs such as MOF-399 exhibit record-low crystal densities around 0.13 g cm^−3^ with void fractions close to 0.94, and the majority of experimental MOFs lie in the ∼0.2–0.8 g cm^−3^ density range with void fractions typically between 0.5 and 0.9.^1,32^ In contrast, dense inorganic frameworks and MXene-based materials routinely reach packing densities of 2.2–4.0 g cm^−3^ or higher.^33^ Our GeoSanity window (density 0.1–3.0 g cm^−3^, void fraction (0, 0.95), generous bounds on PLD/LCD and surface area) is therefore intentionally broad: it flags only extreme, likely unphysical cases while leaving the vast majority of plausible MOFs unflagged. The flags do not replace the anomaly detector; instead, they provide coarse, interpretable labels that help explain *why* a given structure is judged anomalous (*e.g.* clearly unphysical density *versus* subtle topological outliers).

Our ChemSanity flag is conceptually related to recent oxidation-state-based validators such as MOSAEC,^20^ but is intentionally lighter-weight. We rely on a small set of heuristic checks on metal oxidation states, coordination numbers, and charge balance to detect clearly unphysical chemistries, whereas MOSAEC employs a more exhaustive rule set and dedicated atom-typing machinery. In CHEM-AD, these sanity flags are not used to train the autoencoder or to define anomaly scores; instead, they serve as an orthogonal diagnostic for interpreting high-scoring entries and for separating chemically implausible artefacts from structurally unusual yet credible frameworks.

### 2.8. Implementation and reproducibility

All analyses were carried out in Python 3.10 with standard scientific libraries. The complete pipeline—from feature extraction to autoencoder training and visualization—runs in under 15 minutes on a CPU workstation. Code, curated datasets, and result files (CSV anomaly scores) are openly available in the Anomaly_Detection_CHEM_AD repository at https://github.com/alimardani76/Anomaly_Detection_CHEM_AD.

## 3. Results and discussion

### 3.1. Autoencoder convergence and what it tells us

**Fig. 1:**
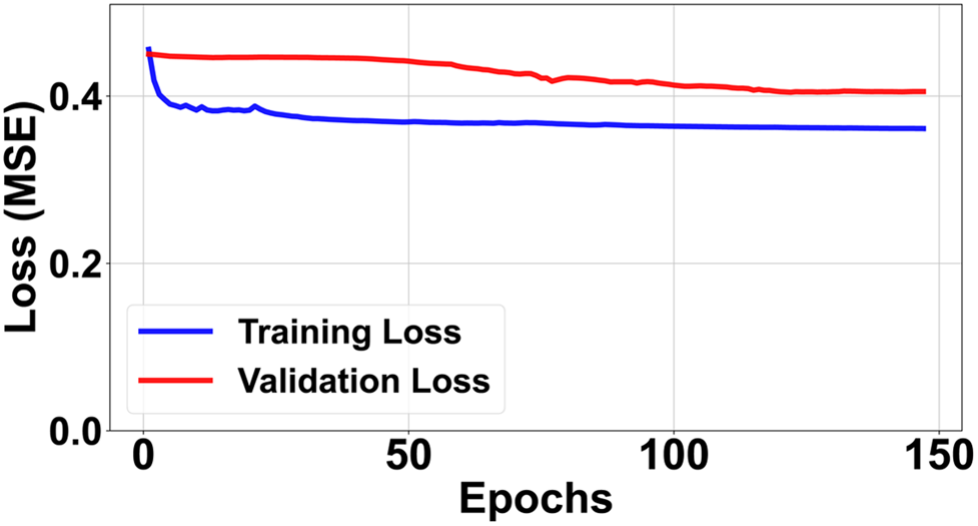
Learning dynamics for the autoencoder trained on standardised descriptors. Training and validation MSE drop rapidly and then evolve into a broad, stable plateau without divergence, indicating that the model has reached a regime of stable compression without strong overfitting. The vertical axis starts at zero to show the full loss scale. Final losses: training 0.3204, validation 0.3954.

Trained on the 81-dimensional MOFxDB descriptor vectors, the symmetric autoencoder converges briskly and then enters a stable regime (Fig. 1). The training and validation losses drop steeply over the first 30 epochs and thereafter evolve into a broad plateau, with the validation curve paralleling the training curve. This behaviour is consistent with effective compression rather than memorisation: autoencoders naturally reconstruct training instances slightly better than unseen samples, so a small and stable gap is expected. Crucially, the validation loss does not diverge but instead remains flat, indicating that the model has learned a compact latent representation of typical MOFs without collapsing or memorising individual descriptors. The architecture is deliberately low-capacity (relative to the dataset size), enforcing a *q*-dimensional bottleneck that acts as a compact manifold for normal structures in descriptor space, while the decoder reconstructs *x̂* from this constrained representation.

We do not interpret the plateau as proof that the loss cannot be further reduced in the non-convex optimisation landscape. Instead, we regard it as a practical indicator of diminishing returns under our chosen optimisation protocol (Adam with weight decay, Batch Normalisation, learning-rate scheduling and early stopping; see Sections 2.3 and S3). In this regime, both the loss curves and our anomaly-separation metric (MDR) become stable across independent train–validation–test splits, which we take as evidence that the resulting anomaly scores are robust to further training. The implementation is lightweight and fully reproducible: training the model completes in approximately 6 minutes on a typical 8-core Intel^®^ Core™ i7 processor. The final standardised mean squared errors (MSE) are 0.3204 for the training set and 0.3954 for the validation set. Although the absolute reconstruction loss lacks direct chemical interpretability, the ranking of MOFs by reconstruction error is stable and meaningful, and it forms the basis for all downstream analyses.

### 3.2. Anomaly-score distribution and thresholding

To quantify how unusual each MOF is, we use the reconstruction error of the autoencoder—specifically, the mean squared error (MSE) in the 81 standardized descriptors—as an anomaly score. This score reflects how well the autoencoder, trained on the full dataset, can reproduce each MOF's original descriptor profile from its compressed latent representation.

**Fig. 2:**
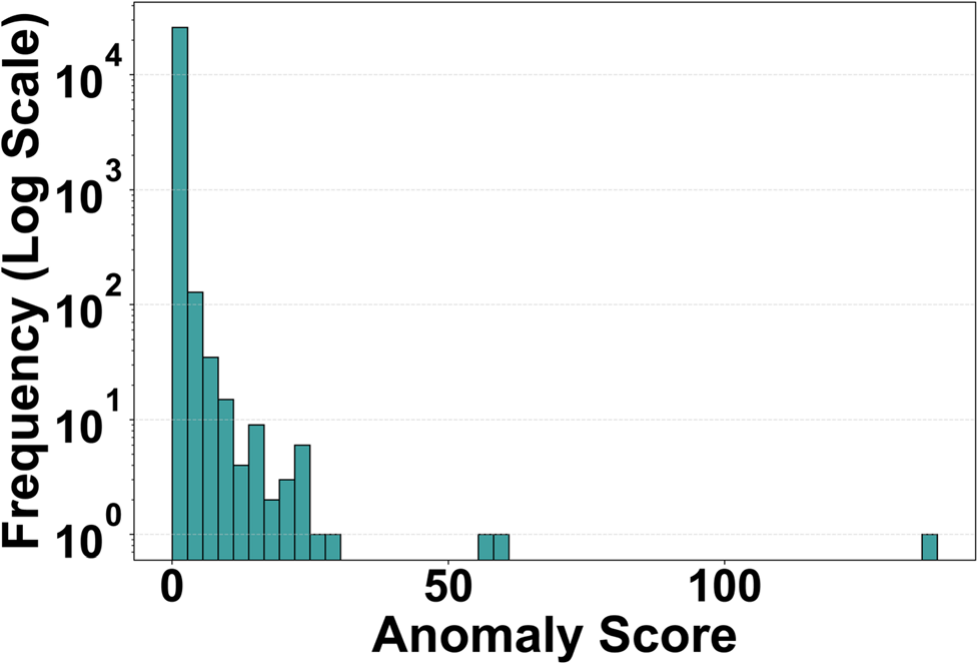
Histogram of reconstruction-error anomaly scores (logarithmic frequency axis). Most MOFs accumulate at low scores, while the log-scaled *y*-axis reveals a sparse but extended right tail corresponding to high-scoring anomalous frameworks with rare descriptor combinations.

When applied to the MOFxDB subset, the resulting distribution of anomaly scores is strongly right-skewed (Fig. 2). The overwhelming majority of MOFs fall within a narrow region of low reconstruction error, meaning their chemical, geometric, and topological descriptor patterns are commonly observed and well-learned by the network. In contrast, a long but thin tail of the distribution contains MOFs with much higher error—indicating they possess unusual combinations of descriptors that are rarely seen together in the dataset, making them harder for the model to reconstruct accurately.

In practical terms, the main body of the histogram represents typical or expected structure–descriptor couplings: examples where the network has learned reliable patterns among pore geometry, connectivity, and composition. The high-scoring tail highlights potential anomalies—MOFs that deviate from these patterns and may correspond to chemically novel structures, mislabeled entries, or data inconsistencies worthy of further investigation.

To convert the continuous anomaly scores into a reproducible classification, we apply a standard method to identify a natural cutoff point—known as the *knee*—on the sorted score curve. Specifically, we use the *kneedle* algorithm,^26^ which locates the point of maximum deviation from the straight line connecting the endpoints of the curve. This knee represents the transition between typical and increasingly atypical structures: beyond this point, each additional MOF admitted into the anomalous group requires a disproportionately higher score.

**Fig. 3:**
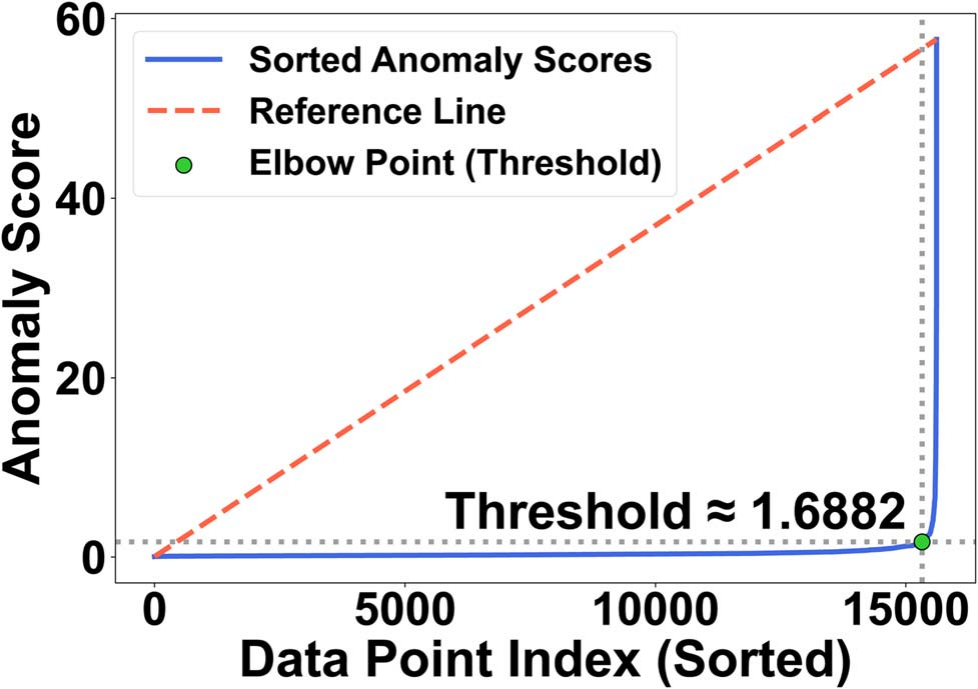
Knee detection on the sorted anomaly scores using the *kneedle* criterion.^26^ The knee at *τ* ≈ 1.6882 (green point) defines the operating threshold, yielding 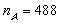 anomalies (∼1.87% of 26 025); higher-scoring MOFs form an increasingly sparse, high-error tail that we inspect in detail in Section 3.4.

For our dataset, the knee occurs at a threshold value of *τ* = 1.6882, resulting in 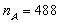 MOFs (∼1.87% of the total 26 025) being labeled as anomalies (Fig. 3).

Closer examination shows that points just above the threshold tend to exhibit minor geometric or topological inconsistencies, such as porosity values outside typical ranges. In contrast, the highest-scoring anomalies also show large MDR—indicating systematic, multi-feature deviations that are more likely to reflect genuine structural novelty rather than noise or data artifacts.

We use this binary split—anomalous 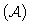
*versus* normal 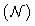
—consistently throughout the analysis. It informs how we visualize structures in descriptor space, compare feature contributions, and organize triage into actionable groups for experimental validation or further curation.

### 3.3. Visualizing anomalies in descriptor space

To interpret how the autoencoder separates typical and anomalous MOFs, we project the learned latent representations into two dimensions using Principal Component Analysis (PCA). PCA preserves global variance structure and provides a linear, easily interpretable view of the 16-dimensional latent space. This makes it better suited than non-linear methods such as t-SNE or UMAP for assessing the overall organisation of normal data and the location of outliers.

**Fig. 4:**
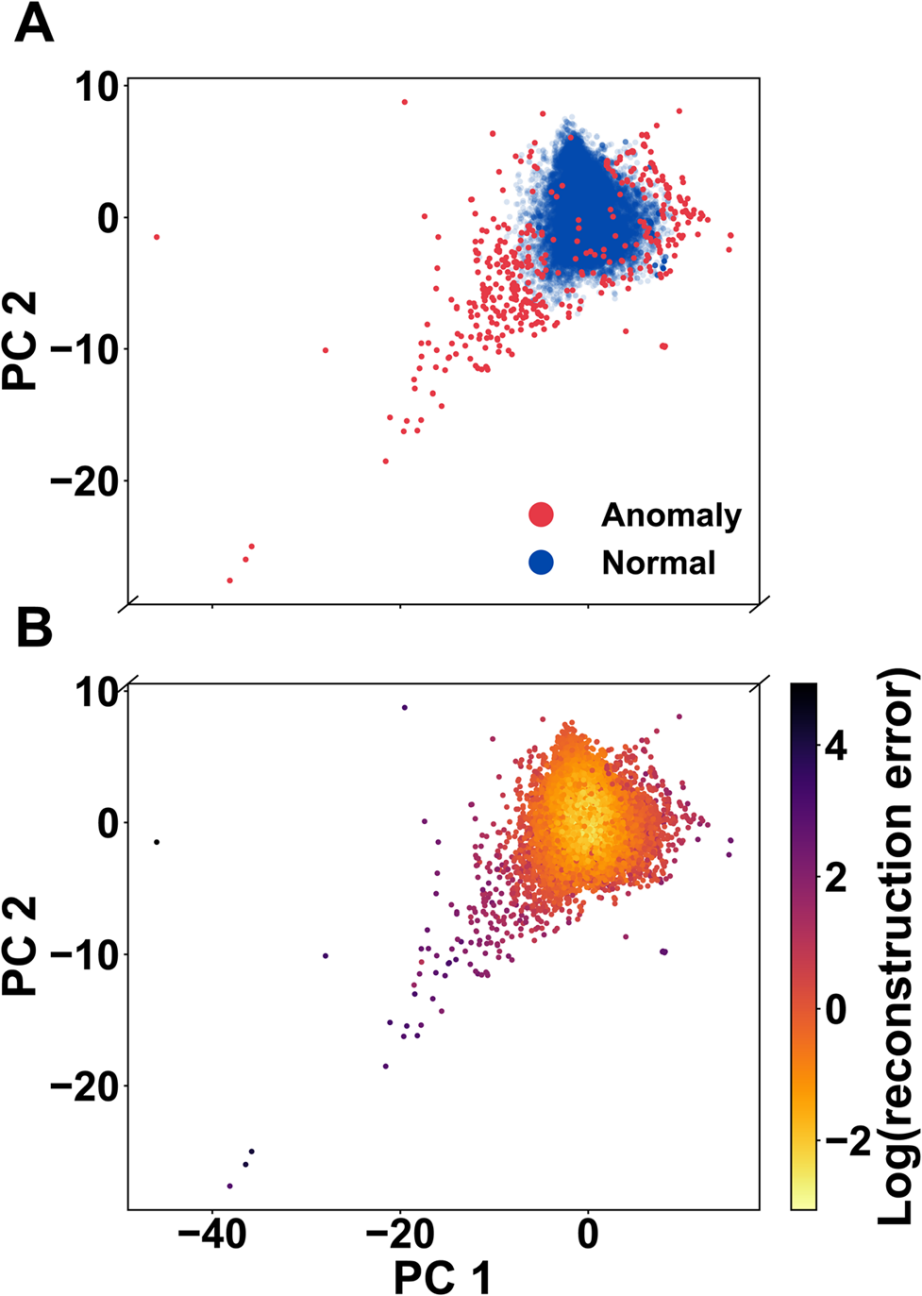
PCA visualisation of the autoencoder latent space. (A) Two-dimensional PCA projection of the 81-dimensional descriptor vectors, with each point representing a MOF coloured by its anomaly label (blue: normal; red: anomalous). Normal MOFs form a dense central cloud, while anomalous structures populate the sparse periphery and tails of the distribution. (B) The same PCA embedding (coloured using a perceptually uniform color map; inferno) by the logarithm of the reconstruction error. High-error MOFs (dark points) again concentrate on the outskirts of the main cloud, whereas low-error structures occupy its dense interior. Together, these views indicate that the autoencoder maps typical MOFs to a compact latent region and places anomalous MOFs in geometrically peripheral, low-density regions.

Fig. 4A shows the PCA projection with points coloured by their anomaly label (blue: normal; red: anomalous). Most MOFs form a dense, coherent cloud, consistent with a compact “normal” manifold in latent space. Anomalous structures, by contrast, populate the sparse periphery and the tails of this distribution rather than the interior of the bulk cluster.

In Fig. 4B, we colour the same PCA embedding by the logarithm of the reconstruction error. High-error MOFs (dark points) again concentrate on the outskirts of the main cloud, while low-error structures occupy its dense core. This concordance between binary anomaly labels and reconstruction-error gradients supports our intended mechanism: the autoencoder maps typical MOFs to a concentrated latent region and consigns unusual, hard-to-reconstruct MOFs to geometrically peripheral, low-density regions.

These peripheral clusters correspond to chemically and structurally distinct frameworks. One group includes ultra-porous MOFs with large cavities but small pore windows (LCD ≫ PLD), yielding unusually high surface areas at low densities. Another group consists of compact, highly connected structures with small pore sizes and high graph density. A third cluster contains frameworks with uncommon coordination features—such as broad node degree distributions, elevated graph transitivity, or nonstandard linker-to-metal ratios. Because such patterns occupy sparsely sampled regions of the CoRE + edge descriptor space, the autoencoder sees few similar examples during training and consequently assigns them larger reconstruction errors.

Conversely, structures with only slightly elevated scores but located within the dense central region of the map often fail simple plausibility checks—such as unphysical coordination numbers or unrealistic porosity—suggesting their anomalies arise from noise or descriptor artifacts. Meanwhile, entries on the periphery that pass all geometric and topological sanity checks tend to have large Mahalanobis distances from the bulk, supporting their classification as genuinely unusual.

These low-dimensional maps provide intuitive visual guides for interpreting model behavior and prioritizing candidates for follow-up analysis. The final anomaly classification, however, is determined exclusively by the quantitative pipeline—autoencoder reconstruction error (thresholded at the knee), plausibility filters, and multivariate distance metrics—to ensure robustness and reproducibility. For completeness, we also examined a t-SNE embedding of the latent space (Fig. S2). Because t-SNE can distort global geometry and produce visually appealing but potentially misleading cluster patterns, we treat it strictly as a qualitative exploratory tool and do not base any quantitative interpretation on it. The PCA projections in Fig. 4 therefore remain the primary method for assessing the global structure of the latent space and the placement of anomalous structures. Although AE score and Mahalanobis distance show a positive trend across the population, but individual points may deviate due to projection effects and because the two metrics quantify different notions of outlierness.

### 3.4. Top-ranked anomalies: triage and interpretation

**Fig. 5:**
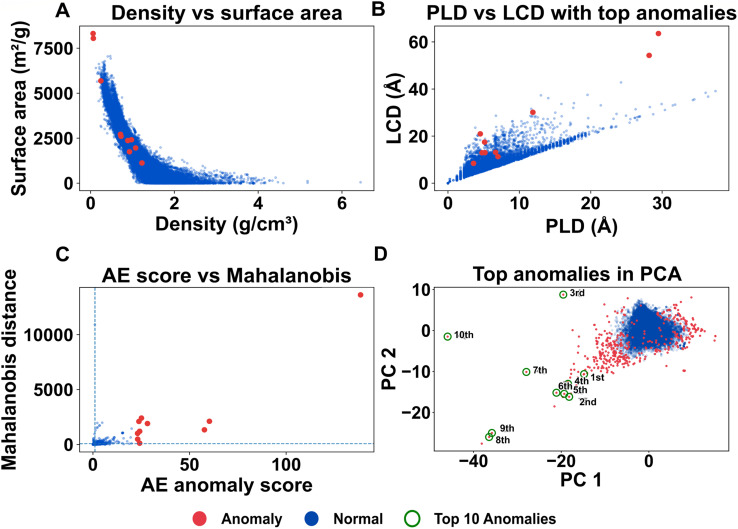
Cross-checks for the ten highest-scoring anomalies. (A) Framework density *versus* gravimetric surface area; top-ranked anomalies (orange markers) lie in sparsely populated, low-density regions of the distribution. (B) PLD–LCD plane highlighting “window-limited” entries whose largest-cavity diameters are two to four times larger than their pore-limiting diameters. (C) Scatter of AE anomaly score *versus* Mahalanobis distance in latent space, showing that high-scoring MOFs tend to have large multivariate distances from the normal population. (D) PCA embedding (same axes as Fig. 4) with the top-10 anomalies highlighted and numbered (1st–10th, green circles). These points lie along sparse filaments and at the periphery of the main cloud, and their ranks match the entries in Tables 1 and 2 and the structures shown in Fig. 6. (Note: apparent 2D distance in this projection does not correspond to Mahalanobis distance, which is computed in the standardized descriptor space.)

**Fig. 6:**
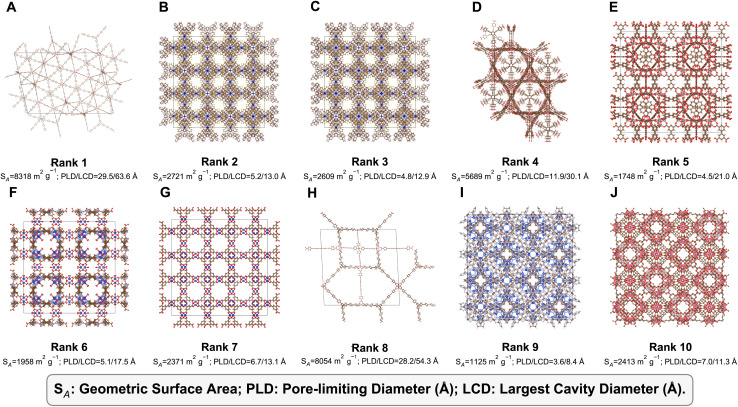
Crystal structures of the ten highest-scoring anomalous MOFs identified by CHEM-AD, ordered by decreasing anomaly score. Panels A–J correspond to ranks 1–10 and are shown as one unit cell with metal nodes (polyhedra) and organic linkers (sticks). For each structure we report the geometric surface area (*S*_A_) and PLD/LCD values (in Å), illustrating the extreme combinations of pore size, density and connectivity that drive the anomaly scores. Ranks 1 and 8 (Class C) exhibit ultra-low densities and very large cavities with high PLD/LCD ratios and multiple sanity-check failures, suggesting database or parsing artefacts. Ranks 2, 3, 4, and 6 (Class B) are geometrically plausible, high-surface-area frameworks with large cages connected by relatively narrow windows, but they carry chemical or topological flags. Ranks 5, 7, 9, and 10 (Class A) are chemically and geometrically reasonable structures whose connectivity and cavity–window geometry place them in sparsely populated regions of descriptor space. Together, these structures illustrate how CHEM-AD surfaces both genuinely unusual, physically plausible frameworks and entries that warrant data-curation scrutiny. All ten structures are experimental entries from the CoRE MOF 2019 subset of MOFxDB; corresponding MOF identifiers are given in Table 2.

Cross-analyses in Fig. 5 and the triage summary in Table 2 show that high autoencoder (AE) anomaly scores arise from specific combinations of geometric and topological descriptors rather than from the sanity flags alone. To make the behaviour of CHEM-AD more tangible, we inspected the ten highest-scoring anomalous MOFs in detail, combining their pore metrics, connectivity features, validation-check outcomes and crystal structures (Fig. 6; rank–ID mapping in Table 1; complete descriptor vectors in Table S2, SI). In all experiments, CHEM-AD is trained and applied to the full mixed dataset described in Section 2.1 (CoRE MOF 2019 + hMOF subset). Interestingly, however, the ten highest-scoring anomalies discussed in this section all originate from the CoRE MOF 2019 subset of MOFxDB, *i.e.* experimentally reported CSD structures rather than hypothetical hMOFs. These ten MOFs span a broad range of geometric properties but fall naturally into three interpretable “triage classes” that reflect how an experimentalist might prioritise follow-up work.

**Table 1:** Mapping from rank to MOF identifier (used only for reproducibility)

Rank	MOF_ID
1	c6ce00407e_c6ce00407e5_clean
2	RIVDIL_clean
3	LAFRAN01_clean
4	c6ce00407e_c6ce00407e6_clean
5	QOYYOU_clean
6	UGOCAW_clean
7	BODPAN_clean
8	c6ce00407e_c6ce00407e4_clean
9	JONKEE_clean
10	HANKOY_clean

**Table 2:** Top-10 anomalous entries (by score) with pore metrics and validation flags

Rank	Anomaly score	Pore metrics			SA (m^2^ g^−1^)	Checks			
		PLD (Å)	LCD (Å)	Density (g cm^−3^)		Chem	Geo	Topo	Build
1	138.5000495	29.4838	63.5937	0.056921	8318.18	Fail	Fail	Fail	Pass
2	60.25304847	5.18013	12.9952	0.711567	2720.59	Fail	Pass	Fail	Pass
3	57.67896639	4.77109	12.9410	0.722977	2609.17	Fail	Pass	Fail	Pass
4	28.2198162	11.8920	30.1208	0.244069	5688.54	Fail	Pass	Fail	Pass
5	25.10115706	4.51928	20.9843	0.928219	1748.33	Pass	Pass	Pass	Pass
6	24.15996109	5.12425	17.4558	1.073998	1957.74	Fail	Pass	Fail	Pass
7	24.14741115	6.66414	13.1498	0.888077	2370.57	Pass	Pass	Pass	Pass
8	23.78127632	28.1736	54.2762	0.063654	8054.44	Fail	Fail	Fail	Pass
9	23.13924877	3.57402	8.42508	1.225688	1125.13	Fail	Pass	Pass	Pass
10	23.12048633	6.99200	11.2813	0.975183	2413.31	Pass	Pass	Pass	Pass

Across the ten structures, two robust patterns emerge in basic descriptor spaces. In the density–surface area map (Fig. 5A), the top-ranked anomalies occupy low-density regions near the periphery of the cloud, rather than the densely populated core of typical MOFs. In the PLD–LCD map (Fig. 5B), many of these entries sit above the main trend, with largest-cavity diameters (LCD) roughly two to four times larger than their pore-limiting diameters (PLD). These elevated LCD/PLD ratios are characteristic of “window-limited” architectures, where large internal cavities are accessed through comparatively narrow windows and are therefore harder for the autoencoder to reconstruct.

For each anomaly, we then explicitly inspected the descriptor profile—density, surface area, LCD/PLD ratios, and connectivity metrics—and overlaid the Chem/Geo/Topo/Buildable flags as an auxiliary diagnostic. In other words, the triage classes in Table 2 are defined primarily by their descriptor signatures, while the sanity flags serve to distinguish physically plausible but unusual structures from entries that exhibit clear indications of database or parsing artefacts.

#### 3.4.1 Class A – structurally plausible, topologically unusual (ranks 5, 7, 9, and 10)

Class A contains four frameworks that pass all geometric and buildability checks and, in three cases, all chemical sanity checks as well. Their densities lie in a physically reasonable range (≈0.89–1.23 g cm^−3^) and their geometric surface areas span roughly 1100–2400 m^2^ g^−1^. Pore metrics are also credible: PLD values between about 3.6 and 7.0 Å and LCD values between about 8.4 and 21.0 Å correspond to microporous to small-mesoporous cavities accessible through moderate windows.

What makes these MOFs anomalous is not any single extreme number but their connectivity patterns. They occupy sparsely populated regions of the topological descriptor space, with atypical combinations of node degrees, ring-size distributions and transitivity. In the PCA embedding (Fig. 5D), Class A entries sit on the periphery of the main cloud rather than in isolated “islands”, consistent with being genuine but under-sampled members of the MOF design space. In several cases, strong interpenetration or multiple intertwined nets cause TopoOK to fail under our single-dominant-network criterion; here this is treated as a signal of topological complexity rather than as a structural error.

Structurally (Fig. 6, ranks 5, 7, 9, and 10), these MOFs often exhibit interpenetrated or highly ornamented network architectures. They feature repeated cage motifs connected in unconventional ways, ring tilings that diverge from canonical nets such as *pcu*, *fcu*, or *soc*, and frameworks where narrow channels intricately thread through larger cavities. Such topological anomalies represent high-value structural phenomena: chemically and geometrically coherent frameworks whose rare connectivities can yield distinctive adsorption or transport behaviors. Comparable phenomena have been reported in several advanced interpenetrated frameworks, where controlled self-assembly, redox activity, and mechanical flexibility together enable responsive sorption mechanisms and pressure-induced phase transitions.^34–36^ In particular, interpenetration has been shown to enhance structural rigidity while simultaneously enabling dynamic “breathing” and gate-opening effects critical for selective gas adsorption and negative gas adsorption transitions.^37,38^ These findings reinforce the hypothesis that the unusual topologies observed here could translate into unique adsorption dynamics, guest-induced flexibility, or selective transport phenomena within confined pore environments.

#### 3.4.2 Class B – geometrically sound with chemical/topological flags (ranks 2, 3, 4, and 6)

Class B comprises four structures with geometries that remain entirely plausible—void spaces, PLD/LCD ratios and densities in the 0.24–1.07 g cm^−3^ range, with surface areas on the order of 2000–5700 m^2^ g^−1^. All four pass GeoSanity and Buildable checks but systematically trip ChemSanity and TopoOK.

Their common motif is large cavities fed by relatively narrow windows. LCD values between ≈13 and 30 Å combined with PLD values of ≈5–12 Å place them well above the bulk LCD–PLD trend in Fig. 5B. These frameworks therefore look like extreme instances of window-limited porosity: a small number of very large cages connected through constricted channels. In such sparse networks, small deviations in metal coordination or linker assignment can easily produce bond-valence or oxidation-state inconsistencies, explaining their chemical flags.

In the structure gallery (Fig. 6, ranks 2, 3, 4, and 6), Class B entries often appear as extended rod- or sheet-based nets stitched into cavernous frameworks. Although ranks 2 and 3 appear visually similar in the unit-cell rendering (Fig. 6), they correspond to the same underlying framework/topology reported in closely related studies. Their separation in the PCA embedding (Fig. 5D) originates from differences in the *derived periodic bonding graph* used to compute the topological descriptors, rather than implying different chemistry. In particular, both structures contain 10 560 atoms (nodes), but rank 3 has 384 fewer inferred bonds (edges) than rank 2 (10 752 *vs.* 11 136), resulting in stronger fragmentation (385 *vs.* 193 connected components) and a smaller largest-connected-component fraction (0.927 *vs.* 0.964). Such differences are consistent with conservative distance-based bond perception interacting with small geometric variations and/or disorder/partial occupancy in the CIF record. We therefore interpret rank 3 as a *representation/curation* anomaly (fragmented graph) rather than a distinct material, and we summarize the key graph diagnostics distinguishing ranks 2 and 3 in the SI (Tables S2–S4 and Fig. S3).

#### 3.4.3 Why Class B fails ChemSanity and TopoOK (and why we do not discard it)

For Class B (ranks 2, 3, 4, and 6), the ChemSanity failure is driven primarily by the linker bond-length dispersion criterion: the linker bond-length standard deviation slightly exceeds our conservative cutoff of 0.4 Å (0.401, 0.403, 0.429, and 0.486 Å, respectively; Table S1), while the bond-length means remain in a plausible range. The TopoOK failure reflects fragmentation of the derived periodic graph (multiple connected components), with rank 6 particularly extreme (largest-connected-component fraction 0.129 with 708 components), and ranks 2–4 showing many small disconnected components despite a dominant component (Table S1). We interpret these flags as curatable diagnostics rather than exclusion criteria: they can arise from conservative bond perception, disorder/partial occupancy, or incomplete structural records, and therefore motivate targeted structural curation (bonding/oxidation-state checks and connectivity clean-up) rather than ruling out geometrically plausible, window-limited frameworks. Notably, the same behavior can occur across multiple CIF entries of the same framework when conservative bond perception interacts with small geometric differences and/or disorder. Also, additional data can be found in the SI.

They are attractive candidates for light curation (*e.g.* charge balance, oxidation states, bond-valence analysis). If their chemistries can be verified, they are natural promotions to Class A.

#### 3.4.4 Class C – likely database artefacts and ultra-porous outliers (ranks 1 and 8)

Class C contains the two most extreme anomalies. Both exhibit ultra-low densities (∼0.06 g cm^−3^), enormous pore sizes (PLD ≈ 28–30 Å, LCD ≈ 54–64 Å) and very high surface areas (>8000 m^2^ g^−1^). They fail all three sanity flags (ChemSanity, GeoSanity and TopoOK) while still being formally Buildable.

These metrics are beyond what is realistically achievable for stable crystalline MOFs and strongly suggest database or parsing artefacts—for example, solvent molecules misinterpreted as part of the framework, supercells, or mis-assigned occupancies. To support this interpretation more concretely, ranks 1 and 8 show multiple, independent red flags that go beyond “large pores” alone (Table S5). Both have extremely low densities (0.0569 and 0.0637 g cm^−3^) together with void fractions ≈0.97 and very large pore sizes (PLD ≈29–28 Å, LCD ≈64–54 Å) and surface areas >8000 m^2^ g^−1^. In addition, their unit cells are exceptionally large (cell volumes 3.3 × 10^5^ to 1.1 × 10^6^ Å^3^), and the derived periodic graphs are highly fragmented (TopoOK = fail, with a small largest-connected-component fraction for rank 1 and multiple connected components for both; Table S5). Finally, both also fail ChemSanity, consistent with bonding/assignment irregularities. Taken together, these independent signatures are characteristic of entries that warrant database curation (*e.g.*misassigned occupancies/disorder, over-expanded cells, or incomplete structure records). We therefore describe ranks 1 and 8 as *likely* artefacts/curation candidates rather than definitively asserting an error without re-refinement against the original deposition record.

In Fig. 6A and G (ranks 1 and 8), they appear as extremely open scaffolds with long linkers and very sparse metal nodes, consistent with over-expanded or partially collapsed representations.

Class C entries are therefore best treated as red flags for data curation rather than targets for materials discovery. They illustrate that CHEM-AD surfaces both scientifically interesting structures (Classes A and B) and entries that warrant closer scrutiny or removal from downstream analyses.

As a sanity check against the experimental literature, we also verified that some of the highest-ranked anomalies correspond to well-known frameworks with recognised extreme pore architectures. For example, the Class A entry ranked 7 is the prototypical HKUST-1 (CSD refcode BODPAN), widely used as a benchmark high-surface-area MOF with large cages connected by narrower windows. CHEM-AD correctly places this structure at the high-surface-area, window-limited edge of the density–surface-area and PLD–LCD distributions (Fig. 5A and B), consistent with its established position at the boundary of experimentally accessible MOF space.

Taken together, the triage in Table 2 and the structure gallery in Fig. 6 show that high anomaly scores are not simply proxies for “large surface area” or “low density”. Instead, CHEM-AD isolates three qualitatively different types of edge-of-manifold behaviour: physically plausible but topologically rare frameworks (Class A), geometrically sound structures with minor chemical/topological inconsistencies (Class B), and likely artefacts with unphysical porosity (Class C). In practice, anomaly scores should therefore be interpreted in conjunction with sanity flags and simple geometric checks, not as a stand-alone yes/no label.

Thus, the triage into Classes A–C is fundamentally feature-driven: Class A collects MOFs whose anomaly scores arise from rare connectivity patterns combined with moderate LCD/PLD combinations, Class B from extreme window-limited porosity plus minor chemical inconsistencies, and Class C from unphysical extremes in density and pore size. The sanity flags simply label whether these feature combinations look chemically and geometrically credible; they do not determine the anomaly scores themselves. A systematic assignment of topological nets (*e.g. via* CrystalNets.jl or ToposPro) for the Class A frameworks would be a natural extension of this work, but is beyond the present scope; here we focus on how their connectivity and pore metrics place them at the edge of the learned descriptor manifold.

We emphasise that CHEM-AD does not treat low or vanishing accessible porosity as evidence that a structure is “not a MOF”. Many experimentally reported frameworks are non-porous or collapse upon activation, and such cases can be scientifically interesting anomalies in their own right. In our triage, entries are assigned to Class C only when lack of porosity co-occurs with clearly unphysical combinations of density, pore metrics and sanity flags; in these cases we interpret the anomalies primarily as potential database or parsing issues (*e.g.* misassigned solvent, supercells, collapsed salts) rather than as novel but adsorption-relevant MOFs.

Importantly, these categories align with the edge-of-manifold behavior observed in the PCA visualizations (Section 3.3 and Fig. 4), reinforcing the conclusion that CHEM-AD surfaces coherent, under-sampled structural motifs rather than random noise.

For transparency and reproducibility, the full rank-to-ID mapping is provided in Table 1, and detailed descriptor vectors for all top-ranked anomalies are available in Tables S1 and S2 (SI).

### 3.5. Comparison of anomalous and typical MOFs

Cross-analyses in Fig. 5 confirm that the highest autoencoder (AE) anomaly scores reflect meaningful structural deviations, rather than being driven by outliers in any single geometric or chemical feature.

For the top-10 anomalous MOFs, we examined these deviations on a per-entry basis rather than only in aggregate. Panels A–C of Fig. 5 show that each high-scoring MOF combines several atypical descriptor values: all lie on the sparse, high-leverage edges of the density–surface-area and PLD–LCD plots and simultaneously exhibit large Mahalanobis distances in the full 81-dimensional space. When we cross-reference these feature deviations with Fig. 6, a consistent picture emerges: high anomaly scores are caused by ultra-low densities with huge cavities (Class C), by window-limited high-surface-area frameworks with very large LCD at modest PLD (Class B), or by topologically complex but chemically reasonable nets with uncommon ring tilings (Class A). The flags are therefore used to interpret which of these feature patterns are physically plausible, not to define the anomalies themselves.

In the density–surface area space (panel A), top-ranked anomalies from Table 2 fall along the high-leverage edges of the data distribution, not within the densely populated core. Similarly, in the PLD–LCD plot (panel B), many of these entries exhibit large cavities (high LCD) accessible through much narrower windows (PLD), consistent with a “window-limited” pore architecture (LCD ≫ PLD).

Panel C illustrates strong agreement between AE anomaly rankings and Mahalanobis distance: most top-scoring anomalies also lie far from the central population under a multivariate distance metric, validating that these MOFs are structurally unusual across several descriptors. Panel D shows the same PCA embedding as Fig. 4, with the ten highest-ranked anomalies highlighted and labelled; all ten occupy the sparse outskirts of the main cloud rather than its dense interior. For reproducibility, the mapping of rank to MOF ID is given in Table 1.

To make these categories more concrete, Fig. 6 presents unit-cell views of the ten highest-scoring anomalous MOFs together with their geometric surface areas and PLD/LCD pairs. The gallery shows that the three triage classes correspond to recognisable structural motifs rather than abstract points in descriptor space.

Ranks 1 and 8 illustrate the ultra-porous extreme. Both frameworks have enormous geometric surface areas (*S*_A_ ≈ 8318 and 8054 m^2^ g^−1^) and unphysically large pores (PLD ≈ 29–28 Å, LCD ≈ 64–54 Å). Visually, they appear as very sparsely connected scaffolds: long linkers span large distances between relatively few nodes, producing huge, weakly supported cavities. Combined with their multiple sanity-check failures, these structures are typical of database artefacts such as over-expanded cells or mis-assigned solvent.

Ranks 2, 3, 4, and 6 correspond to the “window-limited” architectures that sit on the upper envelope of the PLD–LCD plot. They display LCD values in the 13–30 Å range but PLD values of only ≈5–12 Å (*S*_A_ ≈ 1958–5689 m^2^ g^−1^). In the structure panels, this manifests as frameworks built from large cages stitched together by relatively narrow channels or windows. These are geometrically plausible high-surface-area MOFs in which a small number of constrictions control access to very spacious internal cavities—exactly the kind of unusual connectivity that pushes them to the edge of the learned manifold and triggers Chem/Topo flags.

Finally, ranks 5, 7, 9, and 10 are more compact but topologically distinct frameworks. Their surface areas (≈1100–2400 m^2^ g^−1^) and PLD/LCD pairs fall within the envelope of typical microporous MOFs, but they occupy sparsely populated regions of the *topological* descriptor space. In contrast to Class B, these entries typically exhibit TopoOK = pass (*i.e.* a single dominant connected framework component) and distinct combinations of graph metrics (*e.g.* component structure, ring statistics, assortativity and entropy; see Table S6). We therefore use Fig. 6 as an illustrative snapshot, while the triage classes themselves are defined by the computed descriptor signatures rather than by visual inspection.

Together, the structural snapshots in Fig. 6 confirm the story told by the PCA and scatter plots: CHEM-AD is not merely selecting random noisy entries, but systematically surfacing ultra-porous artefacts, window-limited high-surface-area frameworks, and topologically unusual yet physically plausible MOFs as distinct classes of edge-of-manifold structures.

**Fig. 7:**
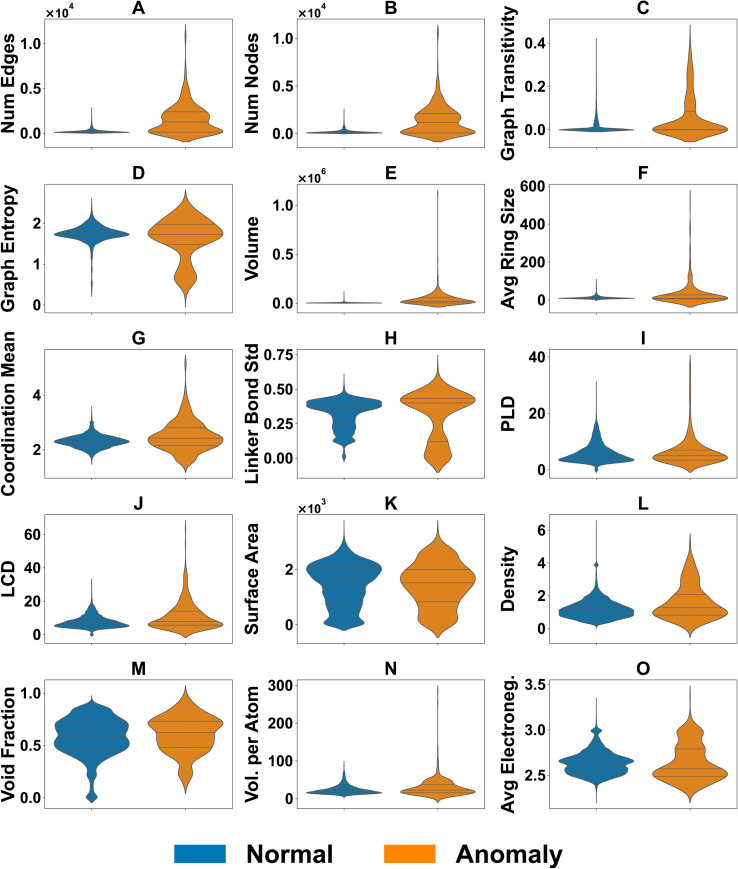
Violin-plot comparison of key descriptor distributions for anomalous (orange) *versus* normal (blue) MOFs identified by CHEM-AD. Panels (A–O) correspond to: (A) number of edges, (B) number of nodes, (C) graph transitivity, (D) graph entropy, (E) framework (unit-cell) volume, (F) average ring size, (G) mean coordination number (coordination mean), (H) linker bond-length standard deviation, (I) pore-limiting diameter (PLD), (J) largest cavity diameter (LCD), (K) geometric (gravimetric) surface area, (L) framework density, (M) void fraction, (N) volume per atom, and (O) average electronegativity. Across these descriptors, anomalous MOFs are enriched in more highly connected graphs (higher node/edge counts) with increased transitivity and entropy, show broader spreads in coordination and linker bond-length variability, and exhibit a systematic tendency toward window-limited pore geometry (larger LCD at comparable or smaller PLD). Consistent with these geometric/topological shifts, anomalies also display heavier high-surface-area tails and widened density/void-fraction and volume-related ranges. Overall, the distributions indicate that the anomaly signal is driven by combined effects of connectivity and cavity–window mismatch, rather than by any single descriptor in isolation.

Now to understand why these MOFs score highly, we compare statistical distributions of key descriptors between anomalous and typical structures (Fig. 7). Anomalies consistently display higher node and edge counts, elevated transitivity, and broader distributions in coordination number and degree centrality. Chemically, they show greater variability in linker bond lengths. Geometrically, these MOFs tend to have larger cavity diameters (LCD) for similar or even smaller PLDs—again indicating limited pore accessibility through narrow windows. Surface-area distributions for anomalies have heavier high-value tails, while density and void fraction are more dispersed but not systematically shifted.

Importantly, no single descriptor defines the anomalous set. Rather, it is the joint occurrence of uncommon connectivity patterns and atypical cavity–window geometries that drives the AE model's high scores. Extended comparisons between anomalous and normal MOFs are provided in Fig. S1 (SI), where porosity-related features (*e.g.*, void fraction, PLD/LCD ratios) and topological descriptors (*e.g.*, ring size, connectivity variance) further illustrate that anomalies represent multi-dimensional deviations, not isolated outliers in a single domain.

These observed patterns are consistent with the structure-level triage in Section 3.4 and the summaries in Table 2. In brief, Class A anomalies (ranks 5, 7, 9, and 10) combine chemically and geometrically plausible features with atypical connectivity and cavity–window geometry: they sit on the edge of the density–surface-area and PLD–LCD distributions without violating our sanity checks, and are therefore high-priority candidates for follow-up studies. Class B (ranks 2, 3, 4, and 6) shares a similar geometric profile but carries systematic ChemSanity and TopoOK flags, reflecting modest coordination or oxidation-state inconsistencies in otherwise plausible, window-limited frameworks. Class C (ranks 1 and 8) gathers the two most extreme entries, with ultra-low densities, very large pores and exceptionally high surface areas, all failing ChemSanity, GeoSanity and TopoOK while remaining formally Buildable; these are best interpreted as database or parsing artefacts rather than as realistic materials. Detailed numerical ranges and structure-level examples for each class are provided in Section 3.4 and Fig. 6.

Across all ten top-scoring entries, GeoSanity and Buildable pass for the majority, whereas ChemSanity and TopoOK are more selective, especially for Classes B and C. This trend underscores that the anomaly detector is primarily sensitive to atypical combinations of network connectivity and pore geometry, not merely to extreme surface areas or densities. The combination of AE anomaly scores and Mahalanobis distance converges on a distinct set of edge-of-manifold MOFs (Fig. 5A–C), while Fig. 7 highlights the corresponding feature shifts—particularly rare combinations of graph connectivity, window–cavity mismatch and framework volume. Full numerical descriptor vectors for each entry are provided in Table S2 (SI).

It is important to emphasise that these flags are high-precision but low-recall diagnostics: they only catch entries that are clearly unphysical. Many chemically and geometrically reasonable yet unusual MOFs pass all flags; the autoencoder assigns them high anomaly scores because of their rare combinations of features in the full 81-dimensional descriptor space. In practice, the flags and the anomaly detector are complementary: the detector ranks suspicious structures, and the flags help distinguish likely artefacts from genuinely interesting, unusual candidates.

### 3.6. Feature-wise contributions to anomaly detection

To better understand what drives the anomaly scores, we compute per-feature contributions using the excess reconstruction error metric Δ_*j*_ (see Section 2.6). For each descriptor *j*, Δ_*j*_ measures how much, on average, anomalous MOFs deviate from the autoencoder's reconstruction along that coordinate. Large values therefore indicate directions in descriptor space where high-score MOFs systematically depart from the learned manifold.

**Fig. 8:**
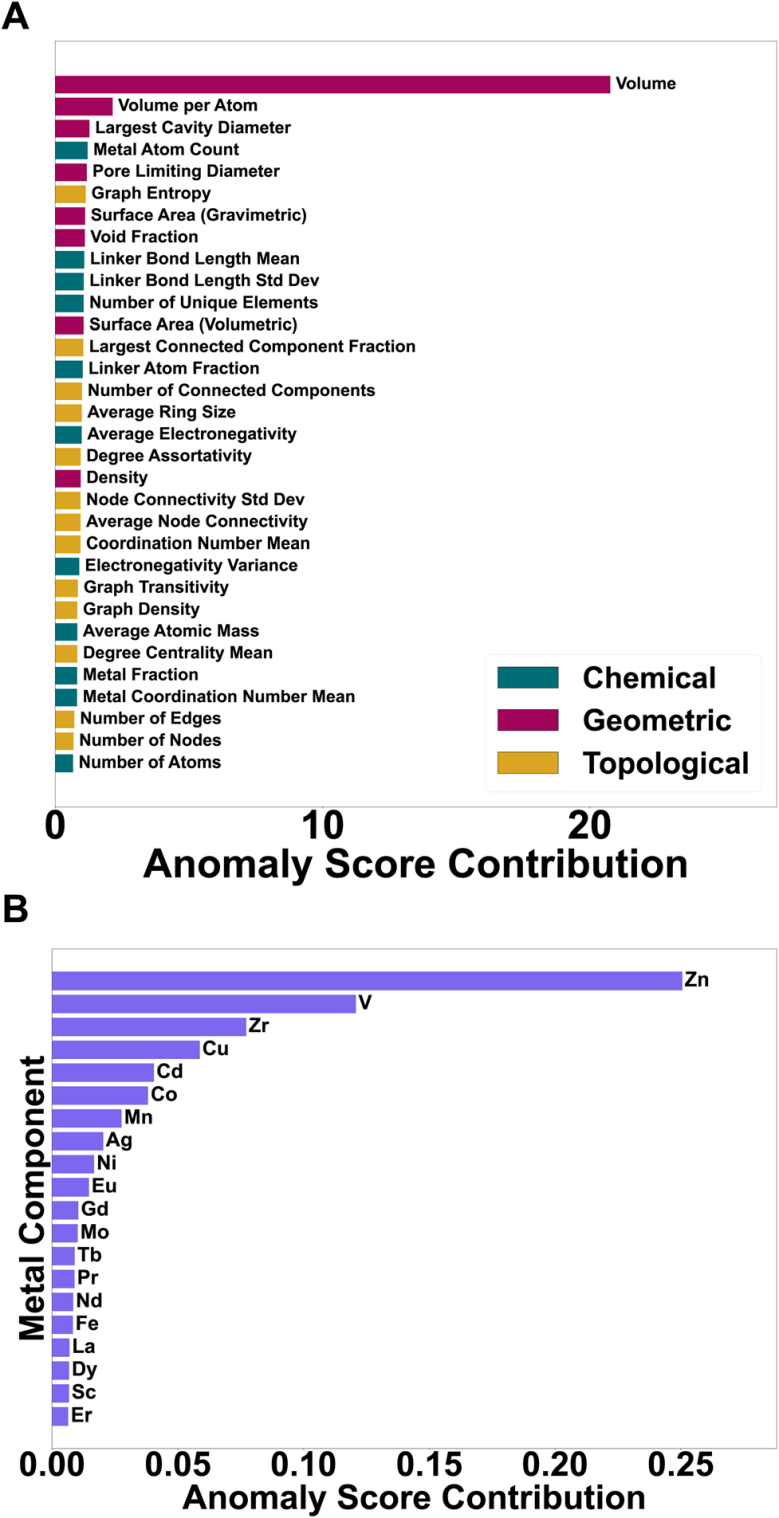
Feature attribution over anomalous MOFs. (A) Per-feature anomaly-score contributions Δ_*j*_ for the 32 baseline descriptors (bars coloured by family: geometric, topological, chemical). Volume-per-atom, LCD/PLD, surface-area and void-fraction terms, together with graph-entropy and connected-component metrics, dominate the signal, whereas atom count contributes weakly. (B) Top 20 contributions of metal-composition features in the composition-augmented model, highlighting metals (*e.g.* Zn, V, Zr) enriched among high-score anomalies.

To avoid biasing the analysis in favour of descriptor families that simply contain more variables, we aggregate contributions at the feature-group level by averaging rather than summing. Let *c*_*j*_ denote the contribution of feature *j* (*e.g.* Δ_*j*_). For a descriptor group *G* (geometry, connectivity, or chemistry) we report the mean per-feature contribution
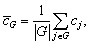
so that a group with many descriptors does not automatically appear more influential than a smaller group. Fig. 8A shows the individual *c*_*j*_ values (bars coloured by family), while the discussion below refers to the corresponding group-averaged trends. We note that *Volume*, *Volume per atom*, and *Density* are classified as geometric descriptors (Section 2.1); and are shown with the corresponding colour in Fig. 8A. Moreover, the strong contribution of *Volume* in Fig. 8A is driven primarily by the small subset of extreme outliers (Class C; ranks 1 and 8), whose unit-cell volumes lie far in the right tail of the dataset. When Class C entries are excluded, the dominant contributors shift toward pore metrics (PLD/LCD, void fraction, surface area) and graph/connectivity measures, confirming that CHEM-AD is not simply a cell-size detector.

#### 3.6.1 Geometric descriptors contribute most strongly, especially *via* pore size and framework volume

In the baseline model, the largest contributions arise from descriptors that jointly encode pore size and framework openness: volume per atom, largest cavity diameter (LCD), pore-limiting diameter (PLD), geometric surface areas (per mass and per volume), and void fraction (Fig. 8A). Together, these quantities describe how much accessible space is available per framework atom and whether that space is concentrated in a few large cavities or distributed more evenly. High-score MOFs are therefore characterised by combinations of very open frameworks (large volume per atom, high surface area, high void fraction) and skewed cavity–window ratios, with LCD/PLD typically about 2–4 (compared with ≈1–1.5 for the bulk of the database). This is consistent with the “window-limited” architectures seen in Fig. 5 and 6, where large cavities are gated by comparatively narrow windows.

#### 3.6.2 Connectivity descriptors identify unusual network organisation

Topology/graph features form the second most influential block. Graph entropy, largest-connected-component fraction, number of connected components, degree assortativity, degree-centrality statistics, and node-connectivity variance all show elevated contributions. These descriptors discriminate between compact, single-component nets and frameworks with supercell-like repetition, partial disconnection, or highly heterogeneous node environments. Their prominence is consistent with the universal TopoOK failures among the top-ranked anomalies (Table 2) and with the peripheral placement of these MOFs in the PCA maps (Fig. 4 and 5D): CHEM-AD is sensitive not only to how much empty space a framework contains, but also to how that space is wired together.

#### 3.6.3 Chemical features modulate, but rarely dominate

Chemical descriptors play a secondary but non-negligible role. Important contributors include the mean and standard deviation of linker bond lengths, the linker-atom fraction, the number of unique elements, and the metal fraction. These indicate that flexible or chemically heterogeneous linkers and mixed-metal environments tend to push structures toward the edge of the manifold. In contrast, bulk elemental averages (mean atomic mass, electronegativity and its variance) contribute modestly, suggesting that the autoencoder is not primarily keying off stoichiometric extremes.

One descriptor that is physically less fundamental is the raw number of atoms in the crystallographic unit cell, since this quantity changes under cell replication while the underlying framework remains the same. In our model this feature acts only as a coarse size-related proxy in combination with density and volume per atom, and its average contribution in Fig. 8A is small compared to invariant quantities such as density, PLD/LCD and the connectivity metrics. Consistent with this, we do not interpret atom count as a primary driver of anomaly scores; the dominant signals come from size-independent geometric and topological descriptors.

To test whether explicit metal identity alters the anomaly landscape, we performed an ablation study in which the baseline descriptor set was augmented with a 49-dimensional multi-hot vector encoding metal composition (SI Section S.4). Fig. 8B summarises the contributions of these composition features. A small subset of metals (*e.g.* Zn, V, Zr) show enhanced contributions, reflecting their enrichment among high-score anomalies, but the overall ranking of anomalous MOFs remains similar: 95.5% of the baseline top-200 anomalies stay within the top-1000 of the composition-augmented model, and the Spearman rank correlation between score lists is *ρ* ≈ 0.59. Explicit composition therefore refines the picture rather than overturning it, shifting emphasis within a chemically consistent subset of already unusual frameworks.

#### 3.6.4 Correlation structure and why the model stays compact

**Fig. 9:**
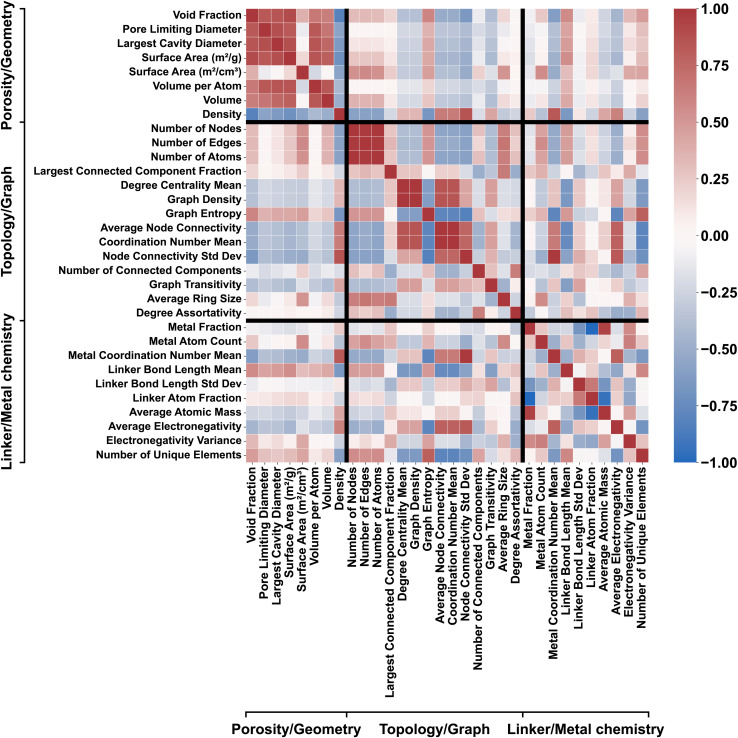
Correlation structure of the 32 baseline descriptors. Pearson correlation matrix showing three main blocks: porosity/geometry (void fraction, PLD/LCD, surface areas, volume, density) anti-correlated with density; topology/graph metrics (nodes, edges, connectivity and entropy); and linker/metal chemistry (metal fraction/count, coordination, linker lengths). Cross-block links (*e.g.* large pores with higher graph entropy and lower graph density) connect geometry to topology and help explain the attribution patterns in Fig. 8.

The correlation heatmap in Fig. 9 clarifies how these attributions arise. The 32 baseline descriptors form three partially redundant blocks: (i) porosity/geometry (surface areas, PLD/LCD, void fraction, volume, density) anti-correlated with density; (ii) topology/graph metrics (nodes, edges, connected-component measures, degree statistics, graph density/entropy); and (iii) linker/metal chemistry (metal fraction and count, mean metal coordination, linker-length statistics). Cross-block links—for example, more open frameworks showing higher graph entropy and lower graph density—connect geometry to topology.

Because the autoencoder compresses strongly collinear descriptors into a low-dimensional latent code, large Δ_*j*_ values identify directions that depart from those correlated blocks rather than double-counting within them. The joint prominence of volume-per-atom and LCD on the one hand, and graph entropy and connected-component statistics on the other, therefore points to a coherent physical picture: CHEM-AD assigns high anomaly scores to MOFs that combine very open, cavity-dominated pore geometry with unusual network organisation and, in some cases, chemically heterogeneous linkers or metals. These are precisely the structures that appear as “edge-of-manifold” MOFs in Fig. 4–6.

It is also useful to place these findings in the context of recent oxidation-state-based validators such as MOSAEC.^20^ Many of the entries gathered in Class C (Section 3.4) exhibit the same kinds of pathologies that MOSAEC is designed to flag—implausible oxidation states, inconsistent coordination environments, or charge-imbalance indicators—and we therefore expect substantial qualitative overlap between Class C and the set of MOSAEC-invalid structures within the CoRE MOF subset. By contrast, most Class A and Class B frameworks pass our ChemSanity checks and would likely be deemed chemically valid by MOSAEC; their high anomaly scores arise from atypical combinations of connectivity and pore geometry rather than from outright chemical errors. A detailed one-to-one benchmark between CHEM-AD anomaly scores, our sanity flags, and MOSAEC labels for CoRE MOFs is a natural direction for future work, but lies beyond the scope of this first study; here we focus on demonstrating that CHEM-AD isolates distinct classes of edge-of-manifold structures that complement rule-based error detectors.

## 4. Conclusion and outlook

We have presented CHEM-AD, a streamlined and accessible anomaly detection framework designed to uncover structurally and chemically unusual metal–organic frameworks (MOFs) from large-scale databases. Using a compact autoencoder trained on 81 standardized geometric, topological, and chemical descriptors, CHEM-AD assigns an anomaly score to each MOF based on its reconstruction error—essentially quantifying how far a structure deviates from the learned norms of the dataset. Applied to 26 025 entries from MOFxDB, this approach identified 488 candidate anomalies, corresponding to approximately 1.87% of the set.

Importantly, these flagged MOFs are not simply random outliers; many display coherent, chemically interpretable features that set them apart. Rather than being defined by a single extreme value—such as unusually high surface area or low density—the anomalies tend to exhibit rare combinations of network connectivity, pore geometry, and compositional features. These include structures with very large internal cavities accessed through narrow windows, frameworks with unusual node and edge statistics, or materials with non-standard coordination environments. These characteristics make them promising leads for further exploration, whether in terms of experimental synthesis, gas adsorption studies, or the development of novel topological classifications.

Beyond highlighting discovery opportunities, CHEM-AD also serves as a valuable tool for dataset curation. Several high-scoring entries exhibit signs of structural inconsistency—such as misparsed solvents, implausible densities, or fragmented topologies—indicating a potential need for reprocessing or manual correction. By separating structurally sound but topologically unusual frameworks from probable data artifacts, CHEM-AD supports more reliable downstream modeling, screening, and machine learning workflows.

The framework organizes results into a simple and reproducible triage. Structures that are topologically uncommon yet chemically and geometrically plausible emerge as high-priority candidates for detailed topological assignment, pore-network tracing, and adsorption simulations. Others may raise minor chemical flags—such as oxidation-state mismatches or charge imbalances—but could be readily validated and elevated to the same level of interest. Entries that exhibit multiple programmatic failures or implausible metrics are likely artifacts and best routed through CIF reprocessing pipelines before interpretation. A current limitation of the present implementation is that it only operates on rows with complete descriptor vectors; entries with missing features are discarded during preprocessing. In future work, CHEM-AD could be extended to handle missing descriptors natively, for example by incorporating mask-aware architectures, robust imputation schemes, or models that operate directly on graphs or raw CIFs without requiring a fixed-length, fully observed feature vector.

Looking ahead, the potential of CHEM-AD extends well beyond MOFs. Its descriptor-based design is inherently portable to other porous material classes, including covalent organic frameworks (COFs), porous polymers, and zeolites. It also offers a pathway toward monitoring shifts in database composition over time—such as those caused by new synthesis trends or curation standards—making it valuable for longitudinal dataset auditing. Moreover, the output of CHEM-AD can serve as a high-confidence filter or seed set for generative models, graph neural networks, or other machine-learning systems aimed at designing new frameworks.

## Author contributions

Conceptualization, M. A.; data curation, S. A.; software, H. A. and S. A.; formal analysis, H. A. and S. A.; validation, H. A. and S. A.; visualization, H. A. and S. A.; methodology, M. A., H. A., and S. A.; writing—original draft, S. A., H. A., and M. A.; writing—review & editing, M. A., S. A., and H. A.; supervision, M. A.; project administration, M. A., H. A. and S. A. contributed equally to this work.

## Conflicts of interest

There are no conflicts to declare.

## Supplementary Material

SC-OLF-D5SC06431G-s001

## Data Availability

The curated descriptor matrix, per-MOF anomaly scores, and per-feature reconstruction-error tables are available at Zenodo: https://doi.org/10.5281/zenodo.17661786 (v2.0). Analysis code and figure notebooks along the codes regarding to reproduce the workflow—including feature-vector generation from raw MOF files—is available in the GitHub repository are available at: https://github.com/alimardani76/Anomaly_Detection_CHEM_AD/. Supplementary information (SI): PDF containing full materials required to reproduce figures and tables in the main text, including: Table S2 (32-descriptor matrix for the top-10 ranked anomalies), Fig. S1 (descriptor distributions for anomalous *vs.* typical MOFs), and Note S1 (descriptor definitions, units, and pass/fail ranges used for ChemSanity, GeoSanity, TopoOK, and Buildable). See DOI: https://doi.org/10.1039/d5sc06431g.
