# Supplementary material for: Decoding the unseen: unsupervised anomaly detection in metal–organic frameworks for discovery beyond the norm
Source: Chem Sci. 2026 Feb 24;17(16):7967–85. doi: 10.1039/d5sc06431g (PMC12952661; doi:10.1039/d5sc06431g)
Supplement: SC-017-D5SC06431G-s001 [file SC-017-D5SC06431G-s001.pdf]

Supplementary Information (SI) for Chemical Science.
This journal is © The Royal Society of Chemistry 2026

Decoding the Unseen: Unsupervised Anomaly Detection in Metal–Organic Frameworks for Discovery Beyond the Norm: Supplementary Information

Hosein Alimardani,[†,§] Shayan Abaei,[‡,§] and Mehrdad Asgari[*,¶]

†*Faculty of Engineering, University of Tehran, Tehran, Iran*

‡*School of Chemical Engineering, Faculty of Engineering, University of Tehran, Tehran, Iran*

¶*Lucy Cavendish College, University of Cambridge, Cambridge CB3 0BU, United Kingdom*

§*These authors contributed equally to this work.*

E-mail: ma2000@cam.ac.uk

S1. Descriptor Matrix and Rank–ID Mapping for the Top-10 Anomalous MOFs

The full descriptor profiles in Table 2 and also second and third rank for some numerical features in Table 3, Table 4, Table 5 and Table 6 (ID mapping in Table 1) show that high anomaly scores arise from *combinations* of geometric, chemical, and topological extremes rather than a single outlying feature.

Table 1: Mapping from rank to MOF identifier (used only for reproducibility).

Rank	MOF_ID
1	c6ce00407e_c6ce00407e5_clean
2	RIVDIL_clean
3	LAFRAN01_clean
4	c6ce00407e_c6ce00407e6_clean
5	QOYYOU_clean
6	UGOCAW_clean
7	BODPAN_clean
8	c6ce00407e_c6ce00407e4_clean
9	JONKEE_clean
10	HANKOY_clean

Table 2: Full numerical descriptor set for the top-10 anomalous MOFs identified by the autoencoder (descriptors as rows, columns are ranks 1–10 in descending anomaly score). Units: density ($g\,cm^{-3}$), surface area ($m^2\,g^{-1}$, $m^2\,cm^{-3}$), PLD/LCD (Å); topological quantities are unitless. See Table 1) for rank→MOF ID mapping.

Descriptor	**1**	**2**	**3**	**4**	**5**	**6**	**7**	**8**	**9**	**10**
num_atoms	3906	10560	10560	5562	5520	3904	4992	1302	5760	3872
volume	1115115.609	265042.2962	260859.55	438839.2349	145722.5659	86767.68211	144767.6308	332388.6771	103870.7217	128886.3449
density	0.056920727	0.711567176	0.722976783	0.244069247	0.928219035	1.073998029	0.888076528	0.063653584	1.225687882	0.975182606
avg_atomic_mass	9.786085346	10.75518973	10.75518973	11.59680699	14.75669513	14.37482525	15.50951385	9.786085346	13.31071733	19.54827355
avg_electronegat.	2.477050691	2.526181818	2.526181818	2.53223301	2.655652174	2.652622951	2.72	2.477050691	2.609333333	2.821652893
electronegat._variance	0.095449366	0.086918149	0.086918149	0.164415402	0.274189792	0.226598038	0.266169231	0.095449366	0.153352889	0.272499747
metal_fraction	0.01843318	0.018181818	0.018181818	0.038834951	0.069565217	0.073770492	0.076923077	0.01843318	0.066666667	0.099173554
num_unique_elements	4	5	5	4	4	5	4	4	4	4
metal_atom_count	72	192	192	216	384	288	384	24	384	384
volume_per_atom	285.4878672	25.09870229	24.7026089	78.89953882	26.39901557	22.22532841	28.99992605	255.2908426	18.03311141	33.28676263
surface_area_m2g	8318.18	2720.59	2609.17	5688.54	1748.33	1957.74	2370.57	8054.44	1125.13	2413.31
surface_area_m2cm3	473.468	1935.93	1886.42	1388.32	1622.7	2102.62	2105.25	512.685	1379.08	2353.41
void_fraction	0.969	0.6832	0.6828	0.9004	0.703	0.744	0.7242	0.9694	0.6092	0.7112
pld	29.4838	5.18013	4.77109	11.892	4.51928	5.12425	6.66414	28.1736	3.57402	6.992
lcd	63.5937	12.9952	12.941	30.1208	20.9843	17.4558	13.1498	54.2762	8.42508	11.2813
linker_atom_fraction	0.98156682	0.981818182	0.981818182	0.961165049	0.930434783	0.926229508	0.923076923	0.98156682	0.933333333	0.925619835
linker_bond_length_mean	1.62093165	1.527709225	1.532116603	1.612782219	1.516885138	1.537879246	1.476109329	1.609979559	1.709935121	1.500393555
linker_bond_length_std	0.422498794	0.400951185	0.403017083	0.429100881	0.376028139	0.485689539	0.359361954	0.424491841	0.454965635	0.324747091

Descriptor	**1**	**2**	**3**	**4**	**5**	**6**	**7**	**8**	**9**	**10**
metal_coord_number_mean	4	6	6	4	4.5	2	4	4	4	3
avg_node_connectivity	2.107526882	2.109090909	2.036363636	2.283351312	2.513043478	2.131147541	2.461538462	2.155145929	2.533333333	2.578512397
avg_ring_size	7.108527132	489.8517555	374.1109185	84.02860697	542.1192661	17.73858921	390.8950564	6	248.8386467	353.9429081
coord._number_mean	2.107526882	2.109090909	2.036363636	2.283351312	2.513043478	2.131147541	2.461538462	2.155145929	2.533333333	2.578512397
deg._assortativity	-0.023050216	0.087868198	0.156927788	-0.174939292	-0.283333333	0.095597937	-0.555555556	-0.084729795	-0.021505376	-0.344055141
deg._centrality_mean	0.0005397	0.000199743	0.000192856	0.000410601	0.000455344	0.000546028	0.000493195	0.00165653	0.000439891	0.00066611
graph_density	0.0005397	0.000199743	0.000192856	0.000410601	0.000455344	0.000546028	0.000493195	0.00165653	0.000439891	0.00066611
graph_entropy	1.698064051	1.667682304	1.681410406	1.713698244	1.8405661	1.767184777	1.738149333	1.669958075	1.158938532	1.404164689
graph_transitivity	0	0	0	0	0	0.173913043	0	0	0	0
largest_cc_fraction	0.151305684	0.963636364	0.927272727	0.994786048	1	0.129098361	1	0.614439324	1	1
node_connectivity_std	0.876798672	1.00313558	0.990199913	0.925432572	0.963374661	0.991362861	0.842650088	0.896597306	0.956846673	1.089029184
num_connected_components	48	193	385	16	1	708	1	9	1	1
num_edges	4116	11136	10752	6350	6936	4160	6144	1403	7296	4992
num_nodes	3906	10560	10560	5562	5520	3904	4992	1302	5760	3872
anomaly_score	138.5000495	60.25304847	57.67896639	28.2198162	25.10115706	24.15996109	24.14741115	23.78127632	23.13924877	23.12048633

Table 3: Selected descriptors distinguishing ranks 2 and 3 (RIVDIL_clean vs LAFRAN01_clean). Values are taken from Table S2.

Descriptor	Rank 2	Rank 3
PLD (Å)	5.180	4.771
LCD (Å)	12.995	12.941
S_A ($m^2\,g^{-1}$)	2721	2609
avg_ring_size	489.85	374.11
largest_cc_fraction	0.964	0.927
num_connected_components	193	385
deg_assortativity	0.088	0.157

Table 4: Flag-triggering descriptors for Class B (ranks 2, 3, 4, 6). Values taken from Table S2

Rank	linker_bond_length_std (Å)	num_connected_components	largest_cc_fraction	Chem/Topo
2	0.400951	193	0.9636	fail/fail
3	0.403017	385	0.9273	fail/fail
4	0.429101	16	0.9948	fail/fail
6	0.485690	708	0.1291	fail/fail

Table 5: Quantitative evidence supporting interpretation of ranks 1 and 8 as likely curation artefacts (values from Table S2).

Descriptor	Rank 1	Rank 8
MOF_ID	`c6ce00407e_c6ce00407e5_clean`	`c6ce00407e_c6ce00407e4_clean`
density ($g\,cm^{-3}$)	0.0569	0.0637
void_fraction	0.9690	0.9694
S_A ($m^2\,g^{-1}$)	8318	8054
PLD / LCD (Å)	29.48 / 63.59	28.17 / 54.28
cell volume ($Å^3$)	1.12×10^6	3.32×10^5
volume_per_atom ($Å^3$)	285.49	255.29
largest_cc_fraction	0.151	0.614
num_connected_components	48	9
ChemSanity / GeoSanity / TopoOK	fail / fail / fail	fail / fail / fail
Buildable	pass	pass

Table 6: Quantitative comparison of Rank 2 (Class B) and Rank 7 (Class A). Values from Table S2

Descriptor	Rank 2 (RIVDIL_clean)	Rank 7 (BODPAN_clean)
ChemSanity / GeoSanity / TopoOK	fail / pass / fail	pass / pass / pass
PLD / LCD (Å)	5.180 / 12.995	6.664 / 13.150
S_A (m^2 g^{-1})	2720.6	2370.6
largest_cc_fraction	0.964	1.000
num_connected_components	193	1
avg_ring_size	489.9	390.9
deg._assortativity	0.088	-0.556
graph_entropy	1.668	1.738

Ultralow-density, hyper-porous case

c6ce00407e_c6ce00407e5_clean exhibits an exceptionally low density ($\sim$0.057 g cm^{-3}) together with very high surface area (8318 m^2 g^{-1}) and void fraction (0.969), plus large PLD/LCD (29.5/63.6 Å). This cocktail produces a descriptor vector far from the "typical" manifold, consistent with its very high anomaly score. Such profiles may reflect truly extreme porosity, but can also be symptomatic of expanded cells, partial-occupancy artefacts, or aggressive solvent-removal assumptions—hence they are prime candidates for manual CIF checks.

Compact, high-surface-area microporous set

Several entries, including UGOCAW_clean, BODPAN_clean, HANKOY_clean and JONKEE_clean, cluster around moderate densities ($\sim$0.89–1.23 g cm^{-3}) with microporous PLD/LCD values in the $\sim$3.6–7.0/8.4–17.5 Å range and substantial geometric surface areas ($\sim$1100–2400 m^2 g^{-1}). Void fractions lie around 0.61–0.74, indicating well-developed but not extreme porosity. Their topological descriptors also trend toward relatively compact, well-connected networks (e.g. degree centrality and graph density above the bulk median, non-zero transitivity for UGOCAW_clean). These frameworks sit at a different corner of descriptor space from the hyper-porous c6ce00407e_c6ce00407e5_clean case, yet are scored anomalous for the same underlying reason: their joint combinations of density, PLD/LCD and connectivity are statistically rare in the training set.

Large-ring and connectivity oddities

RIVDIL_clean shows an unusually large average ring-size metric ($\sim$490) alongside significant geometric porosity (LCD $\sim$13.0 Å, $S_A \approx 2720$ m^2 g^{-1}). Related behaviour appears in LAFRAN01_clean and QOYYOU_clean, which couple large ring-size statistics (average ring sizes $\sim$374–542) with sizeable cavities and high surface areas. In contrast, some anomalies

such as JONKEE_clean carry mid-range densities with relatively large cavities (LCD ∼8.4 Å) but elevated graph-entropy values, suggestive of topological complexity and heterogeneous local environments. Such mixtures of geometric openness with atypical network statistics push these structures into the tail of the reconstruction-error distribution, even though none of the individual descriptors is "astronomical" on its own.

Chemistry–geometry interplay

Variations in metal fraction and average electronegativity—for example, the low metal fraction of c6ce00407e_c6ce00407e5_clean (metal fraction ∼0.018, low average atomic mass) versus the higher metal fractions and heavier compositions of QOYYOU_clean and HANKOY_clean—co-occur with strong shifts in surface area and density. The learned manifold is therefore sensitive to both composition and pore metrics: anomalies tend to exhibit simultaneous deviations in metal content, electronegativity profile, and geometric/topological descriptors. The anomalies thus reflect multi-domain deviation rather than a single-axis outlier.

Because these candidates span qualitatively different regimes (ultra-porous vs. compact high-surface-area microporous frameworks; large-ring topologies; unusual metal/linker composition), they are valuable for (i) data-quality triage (cell standardisation, occupancy checks, duplicate removal) and (ii) hypothesis generation about unusual coordination chemistry and pore architectures. We recommend re-computing descriptors after CIF cleanup (standardised cells, explicit H treatment, removal of crystallographic solvents) and cross-matching with curated references; anomalies that persist are strong targets for deeper mechanistic study or targeted synthesis.

For completeness, the full tables—including (i) total anomaly scores for all 26,025 MOFs, (ii) per-feature absolute reconstruction errors e_{ij} and aggregated contributions α_j for every flagged anomaly, and (iii) the 32-descriptor matrix used for model training—are released on Zenodo (see Data Availability).

S2. Per-Descriptor Distribution Comparisons (Normal vs. Anomalous MOFs)

What Figure S1 shows about anomalies

Figure S1 visualises how MOFs labelled anomalous by the autoencoder differ from the normal population across all chemistry, geometry and topology descriptors used for training.

Geometry and porosity dominate—but not alone

Clear separations appear in void fraction, pore-limiting diameter (PLD), largest-cavity diameter (LCD), and surface-area metrics (panels K–P). Anomalies are over-represented both in ultra-porous, ultra–low-density regimes (high surface area, large PLD/LCD, high void fraction) and in relatively dense, compact networks with small pores. This bidirectional spread explains the long tail of the anomaly-score distribution: structures can end up far from the learned manifold by being either much more open or much more compact than typical MOFs, provided that these geometric deviations occur in uncommon combinations.

Topological signatures are distinctive

Graph-derived features (panels U–AF) also show broader and shifted distributions for anomalous MOFs—especially average ring size, node-connectivity variance, number of connected components, and graph transitivity. Anomalies include both highly modular networks (multiple components, reduced largest-component fraction) and intricate nets with large cycles, higher entropy, and atypical assortativity. The anomaly detector is therefore sensitive to connectivity patterns and network organisation, not just to pore-size statistics.

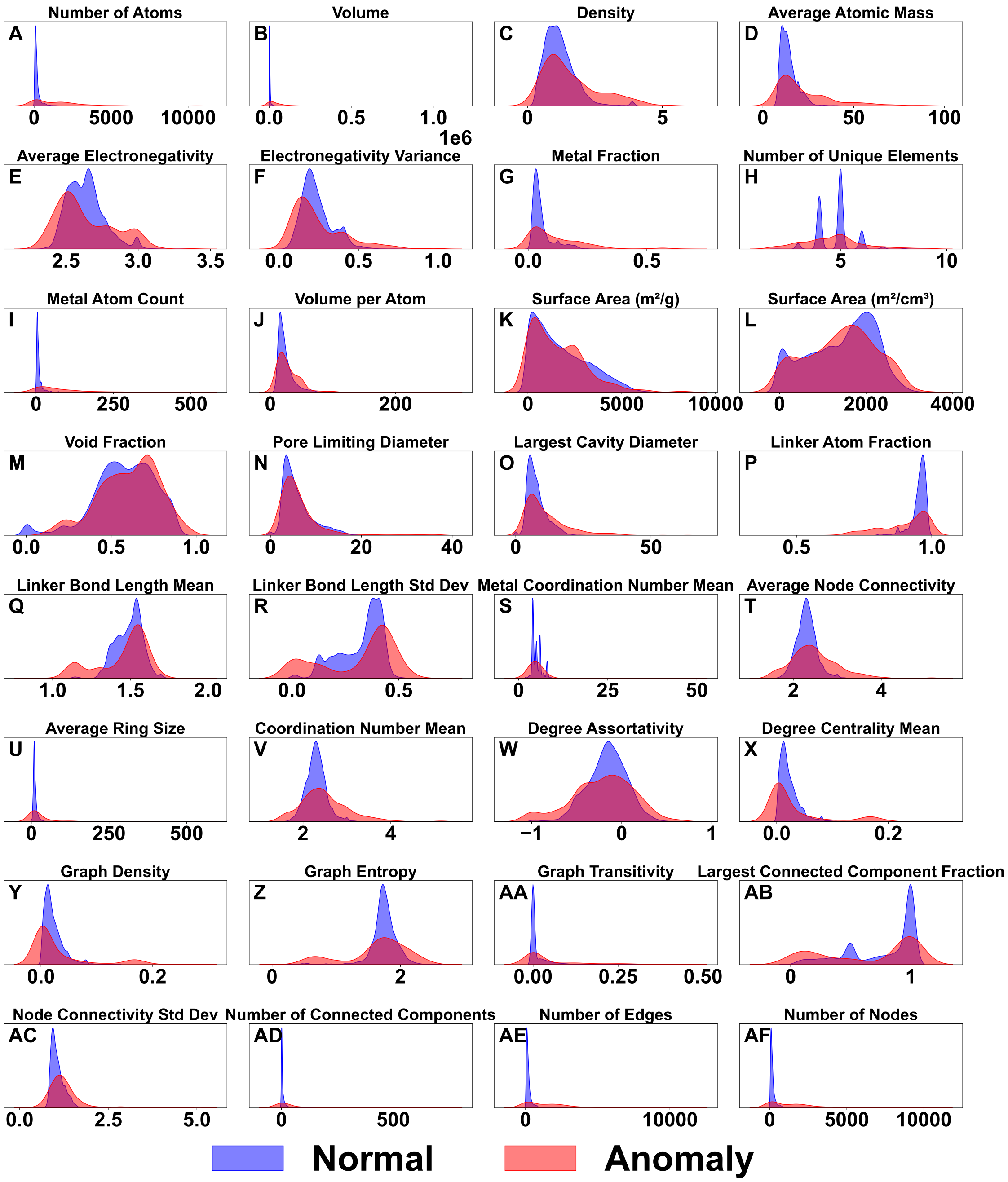

Figure S1: **Descriptor distributions for normal (blue) and anomalous (red) MOFs.** Panels A–AF show kernel-density / histogram overlays for all 32 scalar descriptors used by the autoencoder, grouped as: chemistry (A–J), geometry/porosity (K–P), linker/metal metrics (Q–T), and topology/graph features (U–AF). Blue = normal training population; red = MOFs labelled anomalous ($\text{AnomalyScore}_i > \tau$). Anomalous MOFs populate heavy-tailed, low-density regions of these distributions across multiple descriptor families rather than along a single feature axis.

Chemistry–structure interplay

Chemical descriptors (A–J) provide complementary signals. The anomalous set spans both low metal-fraction systems with extreme porosity and high metal-content frameworks associated with dense, small-pore networks. Shifts in average electronegativity and its variance co-occur with changes in geometric and topological descriptors, consistent with composition constraining feasible coordination environments and preferred network types. The anomalies thus reflect coupled deviations in chemistry, geometry, and topology rather than a purely structural outlier mechanism.

Takeaway for curation and discovery

Because anomalous MOFs populate rare regimes across *multiple* descriptor families, manual follow-up should prioritise (i) CIF standardisation (cell choice, occupancies, crystallographic solvent), (ii) re-computation of descriptors, and (iii) cross-referencing against curated databases. Entries that remain strongly outlying after this cleanup are promising leads for unusual coordination chemistry, nonstandard topologies, or genuinely extreme porosity, rather than artefacts of data processing.

t-SNE embedding (exploratory visualisation)

For completeness, we also computed a two-dimensional t-SNE embedding of the autoencoder latent space (Figure S2). In panel S2A, each point corresponds to a MOF and is coloured by the logarithm of its anomaly score. Panel S2B shows the same embedding with points coloured by their binary label (normal vs. anomalous).

t-SNE is designed to preserve local neighbourhood structure and can therefore give an intuitive view of how nearby MOFs are organised in latent space. However, it is well known to distort global geometry and to produce visually striking but potentially misleading cluster patterns. For this reason, we treat Figure S2 as a qualitative, exploratory visualisation only and do not use it for any quantitative conclusions. All interpretation of the global organisation of normal and anomalous MOFs is based on the PCA projections shown in Figure 4 of the main text.

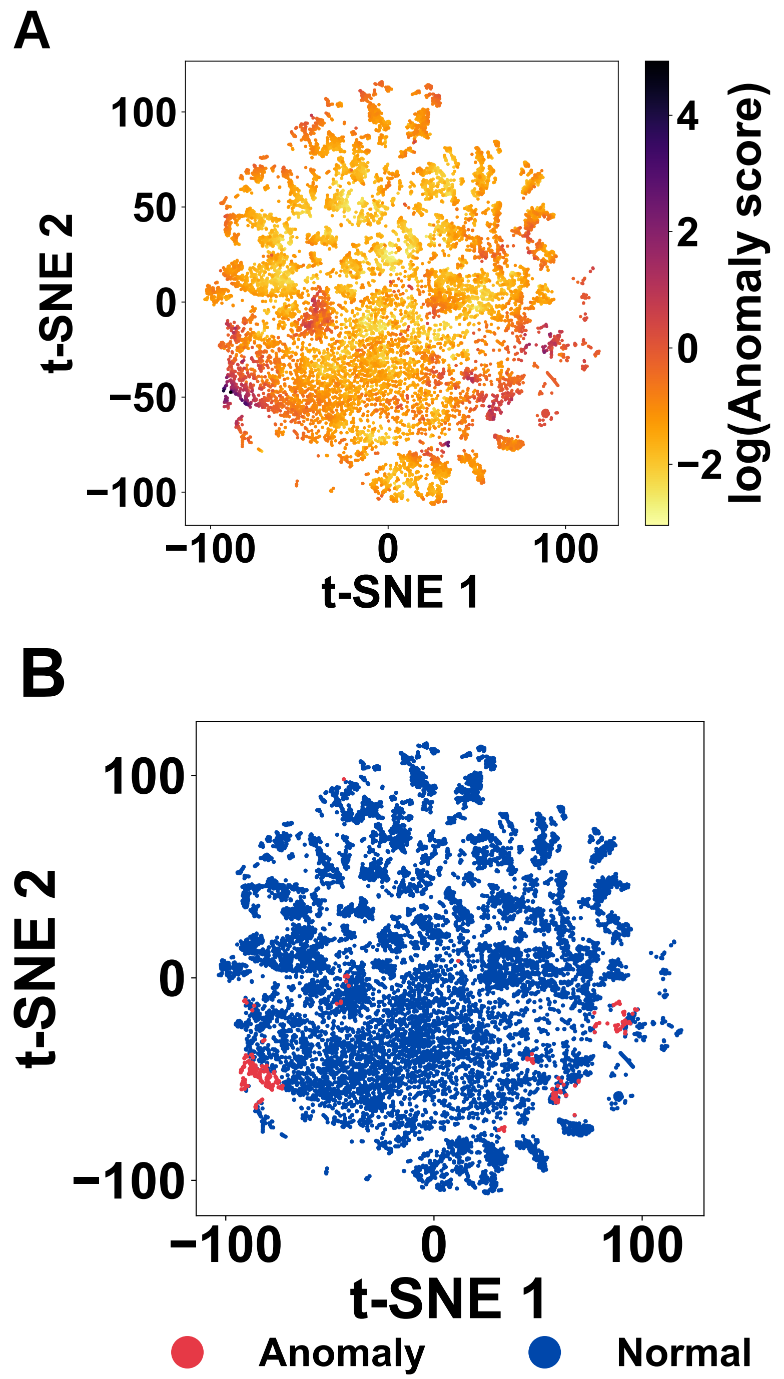

Figure S2: t-SNE embedding of the autoencoder latent space (exploratory visualisation). (A) Points coloured by the logarithm of the anomaly score log(AnomalyScore). (B) The same embedding coloured by the binary anomaly label (red: anomalous; blue: normal). t-SNE emphasises local neighbourhood relationships but can strongly distort global geometry; this figure is therefore provided only as a qualitative illustration. All quantitative interpretation of latent-space structure and anomaly separation relies on the PCA projections in Figure 4 of the main text.

S3. Autoencoder architecture and training

Convergence Strategy

Standard optimization of deep autoencoders can be susceptible to premature convergence, where the optimizer is trapped in local minima or saddle points manifested as plateaus in the validation loss. To ensure robust convergence to a meaningful minimum, we implemented a comprehensive training protocol designed to effectively navigate the complex loss landscape.

The protocol incorporates L2 weight decay (=0.001) to regularize the model and smooth the loss surface, mitigating overfitting and improving the optimizer's path. Furthermore, Batch Normalization is applied after each dense layer to stabilize training and ensure a consistent gradient flow. To actively navigate and escape potential plateaus, an adaptive learning rate strategy was employed via the `ReduceLROnPlateau` callback. This mechanism reduces the learning rate when validation loss stagnates, enabling a more fine-grained search for the minimum. Finally, the `EarlyStopping` callback terminated training when no improvement was observed on the validation set, restoring the model weights from the best-performing epoch. This multi-faceted strategy ensures that a loss plateau is not a terminal condition but a signal to refine the optimization process, guiding the model toward a more generalizable solution.

Architectural Optimization for Anomaly Detection

Our primary objective was to develop an autoencoder architecture optimized for unsupervised anomaly detection. In this context, the model's success is not measured by its ability to accurately reconstruct inputs, but by its capacity to create a clear separation between normal and anomalous data. To quantify this separation, we used the Mahalanobis Distance Ratio (MDR) as our key performance metric. A high MDR indicates that the anomalies identified by the model are distinctly different from the normal data cluster. Our

methodology involved a systematic grid search over two key hyperparameters: network depth (controlled by `tapering_size`) and bottleneck size (the `latent_dim`).

The Flaw of Prioritizing Reconstruction Accuracy

A common approach in autoencoder design is to minimize reconstruction error. However, our findings confirm this is not a viable strategy for anomaly detection. Models that are too flexible learn are adaptable are unable to distinguish between normal and abnormal data.

A clear example is the model with a `tapering_size` of 16 and a `latent_dim` of 64. This shallow and unconstrained architecture achieved an extremely low mean test error of 0.0138. Yet, it failed at the primary task, producing a poor MDR of only 3.1088. This demonstrates that minimizing reconstruction error can be misleading and ultimately detrimental to the goal of identifying anomalies.

The Critical Role of Network Depth and Bottleneck Size

Our analysis revealed a clear, two-stage process for identifying the optimal architecture.

First, network depth proved to be a foundational factor. Deeper networks, created by using a smaller `tapering_size` (e.g., 1 or 2), consistently outperformed their shallower counterparts. These deeper models are better equipped to learn the complex, non-linear patterns that define the "normal" data, creating a more robust foundation for the model.

Second, for these effective deep networks, the size of the informational bottleneck (`latent_dim`) was the most critical factor influencing performance. The autoencoder is forced to compress all information about an input through this bottleneck. This pressure is essential for effective anomaly detection.

- When the bottleneck is too permissive (large `latent_dim`), the model can easily pass through enough information to reconstruct anything, including anomalies. This

leads to poor separation, as seen with the `tapering_size`=2, `latent_dim`=64 model, which had a low MDR of 4.3853.

- As the latent bottleneck narrows, the autoencoder is forced to encode only the most important features of the normal data. This compression prevents the model from memorizing anomalies, making it more sensitive to inputs that deviate from learned patterns. Consequently, the Mahalanobis distance–based separation improves as the bottleneck tightens, up to the point where over-compression begins to degrade normal sample representation.

This trend is clearly visible in the results for the `tapering_size`=2 models. The MDR climbs from 4.9573 at `latent_dim`=2 and peaks with an MDR of 5.4975 at `latent_dim`=16.

Identifying the Optimal Architecture: Balancing Performance and Stability

While the goal is to maximize the MDR, model stability is also crucial for reliable performance. The highest average MDR in our study (6.0802) was achieved by the model with `latent_dim`=32 and `tapering_size`=1. However, this performance was highly erratic, with an extremely large standard deviation of ± 1.9507, making it an unreliable choice.

Therefore, the optimal architecture was identified as the one providing the best balance of high performance and stability. The model with a `tapering_size` of 2 and a `latent_dim` of 16 emerged as the clear winner. It achieved an excellent mean MDR of 5.4975 while maintaining a much lower standard deviation of ± 0.5107. This configuration is powerful enough to create a distinct boundary between normal and anomalous data and is stable enough to do so consistently, making it the most suitable choice for this anomaly detection task.

Table 7: Architectures by Performance. The Mahalanobis Distance Ratio (MDR), Test Error, and Validation Loss columns report the mean ± standard deviation across 5 folds.

Latent Dim.	tapering size	MDR (95%) ↑	Test Error ↓	Validation Loss ↓
1	1	5.1836 ± 0.3923	0.4015 ± 0.0213	0.4132 ± 0.0223
1	2	5.091 ± 0.3944	0.4047 ± 0.0292	0.411 ± 0.0258
1	4	4.8493 ± 0.8336	0.3817 ± 0.051	0.3878 ± 0.0319
1	8	5.1752 ± 0.3669	0.4096 ± 0.0305	0.4186 ± 0.0223
1	16	4.3307 ± 1.0693	0.2391 ± 0.0533	0.2479 ± 0.0594
1	32	4.1619 ± 0.6342	0.2306 ± 0.0175	0.2366 ± 0.0164
1	64	4.3166 ± 0.6607	0.2622 ± 0.0177	0.2694 ± 0.0188
2	1	5.1944 ± 0.3742	0.4064 ± 0.0305	0.4134 ± 0.0228
2	2	4.9573 ± 0.4479	0.4061 ± 0.0316	0.4296 ± 0.0486
2	4	5.2935 ± 1.5600	0.3792 ± 0.0281	0.3845 ± 0.0157
2	8	4.1817 ± 1.0673	0.3115 ± 0.1029	0.3145 ± 0.1051
2	16	3.2505 ± 0.4150	0.135 ± 0.0163	0.1404 ± 0.0163
2	32	4.0203 ± 0.6156	0.1601 ± 0.0146	0.1665 ± 0.0135
2	64	4.3853 ± 0.6982	0.1948 ± 0.0207	0.2003 ± 0.0139
4	1	5.525 ± 0.937	0.404 ± 0.0298	0.5099 ± 0.235
4	2	5.16 ± 0.3481	0.400 ± 0.0221	0.4091 ± 0.0202
4	4	4.4068 ± 0.9713	0.3296 ± 0.0744	0.3343 ± 0.0567
4	8	3.1412 ± 0.5239	0.1632 ± 0.0249	0.1645 ± 0.0327
4	16	2.5852 ± 0.2203	0.0801 ± 0.0054	0.0831 ± 0.0040
4	32	3.7566 ± 0.3919	0.0916 ± 0.0073	0.0952 ± 0.0079
4	64	4.7151 ± 0.6676	0.1186 ± 0.0120	0.1249 ± 0.0117
8	1	5.1164 ± 0.3236	0.4045 ± 0.0285	0.4250 ± 0.0468

Continued on next page

Table 7 – continued from previous page

Latent Dim.	tapering size	MDR (95%) ↑	Test Error ↓	Validation Loss ↓
8	2	5.2656 ± 0.4856	0.4354 ± 0.1021	0.4089 ± 0.0191
8	4	4.1274 ± 0.7802	0.3242 ± 0.0634	0.3306 ± 0.0415
8	8	2.9843 ± 0.3890	0.1501 ± 0.0468	0.1614 ± 0.0726
8	16	2.8322 ± 0.2697	0.0601 ± 0.0048	0.0621 ± 0.0045
8	32	3.4086 ± 0.5293	0.0576 ± 0.0035	0.0606 ± 0.0082
8	64	5.0874 ± 0.8234	0.0808 ± 0.0117	0.0856 ± 0.0123
16	1	5.1203 ± 0.4505	0.4094 ± 0.0242	0.4157 ± 0.0196
16	2	5.4975 ± 0.5107	0.3918 ± 0.0173	0.4042 ± 0.0231
16	4	4.3131 ± 0.7587	0.3404 ± 0.0291	0.3534 ± 0.0354
16	8	2.627 ± 0.2535	0.0963 ± 0.0079	0.1026 ± 0.0196
16	16	2.8996 ± 0.2406	0.0567 ± 0.003	0.0592 ± 0.0056
16	32	3.5236 ± 0.4063	0.0525 ± 0.0073	0.0549 ± 0.0051
16	64	4.838 ± 0.5635	0.0605 ± 0.0085	0.0651 ± 0.0107
32	1	6.0802 ± 1.9507	0.397 ± 0.0259	0.4001 ± 0.0168
32	2	4.9217 ± 0.6435	0.4003 ± 0.0327	0.409 ± 0.0135
32	4	2.7115 ± 0.3891	0.1582 ± 0.018	0.1649 ± 0.0176
32	8	2.9311 ± 0.2769	0.0561 ± 0.0019	0.0587 ± 0.0065
32	16	3.1822 ± 0.3468	0.0355 ± 0.0019	0.0378 ± 0.0052
32	32	3.3407 ± 0.1565	0.0201 ± 0.002	0.0224 ± 0.0038
64	1	5.1779 ± 0.3872	0.4088 ± 0.0306	0.4259 ± 0.0215
64	2	2.7685 ± 0.4648	0.063 ± 0.0044	0.0644 ± 0.005
64	4	3.0636 ± 0.5339	0.0291 ± 0.0017	0.0308 ± 0.003
64	8	3.0507 ± 0.4386	0.0192 ± 0.0016	0.0205 ± 0.0025

Continued on next page

Table 7 – continued from previous page

Latent Dim.	tapering size	MDR (95%) ↑	Test Error ↓	Validation Loss ↓
64	16	3.1088 ± 0.3385	0.0138 ± 0.0017	0.0149 ± 0.0022

Ultimately, to ensure that our findings are robust and not an artifact of a single "lucky" architecture, we analyzed the consistency of the anomalies identified across the hyperparameter landscape. We measured the pairwise percentage overlap of the top-100 anomalies identified by the top 10 models (ranked by MDR). The overlap ranged from 82% to 100%, indicating a strong consensus on the specific structures identified as anomalous regardless of minor architectural variations. In all cases, the feature scalers and reconstruction-error threshold are fitted exclusively on the training data; the MDR is computed on the held-out validation or test sets, and no information from these sets is fed back into the training or calibration steps.

PCA variance profile

The provided data shows the cumulative explained variance for a PCA with 81 principal components (PCs). The variance accumulates rapidly at the beginning: the first component explains 22.94% of the variance, and the first 14 components together capture 90.51%. To retain 95.39% of the variance, 19 components are needed.

Table 8: Cumulative Explained Variance (Top 30 Components)

PC	Var. (%)	PC	Var. (%)
1	22.94	16	93.00
2	40.48	17	93.92
3	54.07	18	94.77
4	60.87	19	95.39
5	66.11	20	95.96
6	70.22	21	96.46
7	74.12	22	96.90
8	77.45	23	97.31
9	80.42	24	97.69
10	82.96	25	98.04
11	85.27	26	98.36
12	87.24	27	98.57
13	89.02	28	98.76
14	90.51	29	98.93
15	91.81	30	99.07

S4. Ablation study on explicit metal-composition features

To quantify the impact of adding explicit metal-composition information, we performed a controlled ablation study. We compared anomaly-score rankings obtained from two autoencoder models that share the same architecture and training protocol but differ in their input descriptor sets:

- **Model A (baseline):** the original 32-dimensional descriptor set, comprising geometric/porosity, aggregate chemical, and topological descriptors as described in Section 2.1 of the main text.
- **Model B (composition-augmented):** the same 32 descriptors, augmented by a 49-dimensional multi-hot metal-composition vector.

The composition vector encodes the *presence or absence* (1/0) of each metal element observed in MOFxDB: Sc, Ti, V, Cr, Mn, Fe, Co, Ni, Cu, Zn, Y, Zr, Nb, Mo, Tc, Ru, Rh, Pd, Ag, Cd, Hf, Ta, W, Re, Os, Ir, Pt, Au, Hg, Al, Ga, In, Sn, Pb, Bi, La, Ce, Pr, Nd, Sm, Eu, Gd,

Tb, Dy, Ho, Er, Tm, Yb, and Lu. We use a binary encoding rather than raw counts because overall atom counts and metal fractions are already captured by existing descriptors; the composition vector is meant to inject *identity* information, not duplicate size-related features.

For this ablation, the input features were partitioned into (i) *numeric descriptors* (all original 32) and (ii) the *multi-hot composition vector*. Only the numeric descriptors were standardized (z-score normalization). The multi-hot vector was kept in $\{0, 1\}$ to avoid imposing spurious ordinal relationships between different metals. Both models were trained with the same autoencoder architecture and hyperparameters as described in Section 2.2 of the main text.

We compared the anomaly-score rankings of Model A and Model B using three complementary metrics:

1. **Global rank stability (Spearman ρ).** The Spearman rank correlation between the two anomaly rankings was $\rho \approx 0.59$ ($p < 0.001$). This moderate positive correlation indicates that the global structure of the anomaly landscape is largely preserved: metal–organic frameworks (MOFs) that Model A flags as anomalous tend, in general, to receive high anomaly scores under Model B as well. This supports the conclusion that the original descriptor set already carries a substantial and meaningful anomaly signal.

2. **Top-K set overlap (Jaccard index).** We examined the overlap between the top-K anomalies from both models using the Jaccard index

$$J_K = \frac{|A_K \cap B_K|}{|A_K \cup B_K|},$$

where A_K and B_K denote the sets of the K highest-scoring anomalies under Models

A and B, respectively. We obtained

$$\text{Jaccard@}K = 50 = 0.49,$$
$$\text{Jaccard@}K = 100 = 0.45,$$
$$\text{Jaccard@}K = 200 = 0.39.$$

These low-to-moderate overlaps show that, although the two models broadly agree on which regions of descriptor space are anomalous, the *specific* structures promoted into the very top of the ranking change substantially when explicit metal composition is included. Model B reorders and enriches the top-K list rather than simply reproducing Model A's top anomalies.

3. **Top-K set containment.** To test whether Model B discards anomalies identified by Model A, we measured the fraction of Model A's top-200 anomalies that appear anywhere in Model B's top-1000. We found that 191 out of 200 entries (95.5%) are retained within this expanded set. Thus, the baseline anomalies are not "lost" when composition is added; many are simply demoted from the very top of the list while still remaining highly ranked.

Interpretation. Taken together, these metrics show that explicit metal-composition features act as a *refinement* of the anomaly signal. The high containment and moderate Spearman correlation indicate that the baseline model was not fundamentally misidentifying anomalies: most high-scoring structures in Model A remain anomalous under Model B. At the same time, the relatively low Jaccard indices demonstrate that Model B consistently promotes additional, chemically expressive outliers—entries whose anomaly is driven in part by unusual combinations of metals—into the very top of the ranking.

In summary, augmenting the descriptor set with a metal-composition vector sharpens the anomaly ranking and helps prioritise compositionally unusual or chemically implausible MOFs, while preserving the core conclusions drawn from the original model.

S5: Graph-Descriptor Sensitivity to CIF-Level Bond Perception and Structural Fragmentation

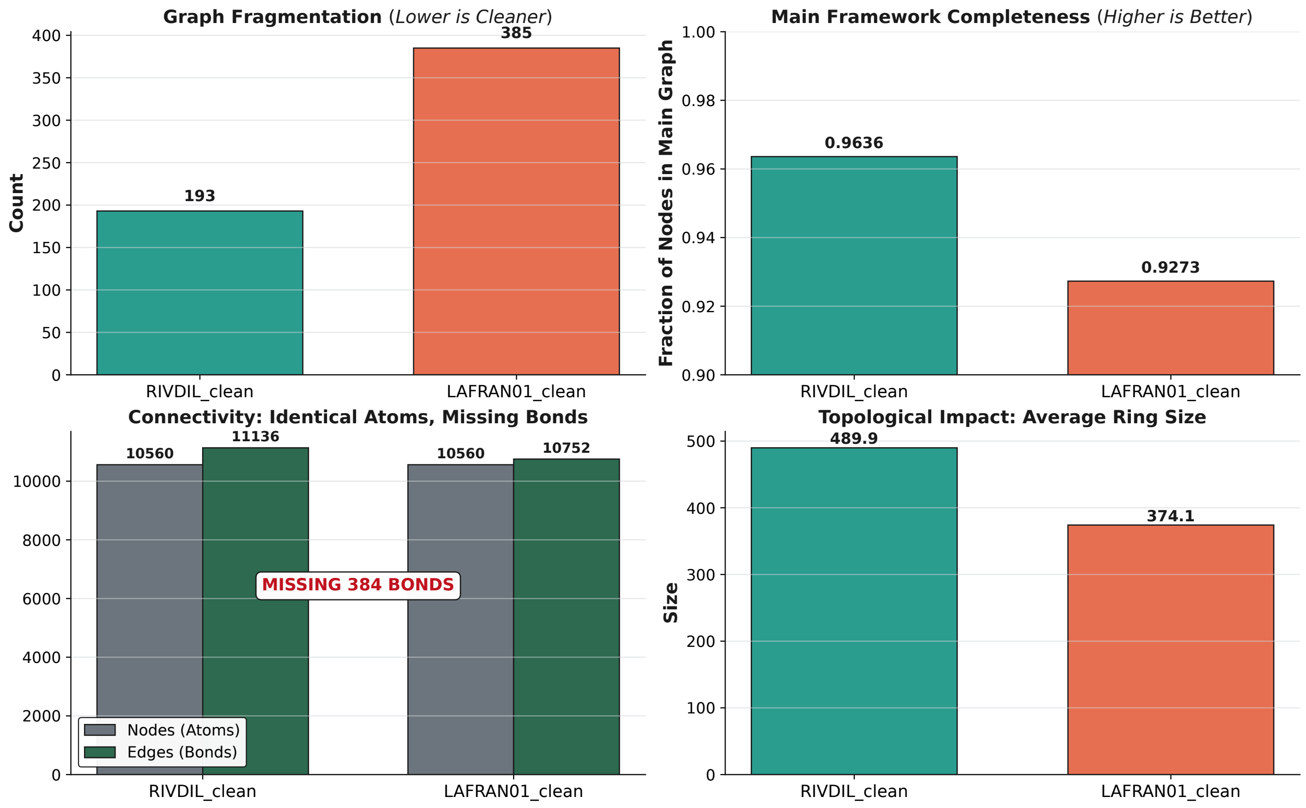

Figure S3: Graph diagnostics comparing ranks 2 and 3 (RIVDIL_clean vs LAFRAN01_clean), which correspond to the same underlying framework but yield different derived periodic bonding graphs under conservative bond perception. Although both entries contain the same number of atoms (10,560 nodes), rank 3 has fewer inferred bonds (10,752 vs 11,136; $\Delta = 384$), leading to increased fragmentation (more connected components), a reduced largest-connected-component fraction, and shifted ring statistics. These differences reflect CIF-level representation/disorder sensitivity rather than distinct chemistry.

Sensitivity of graph descriptors to CIF-level bond perception. The periodic graphs used to compute topological descriptors are generated using a conservative, distance-based bond-perception scheme. For duplicate or closely related CIF entries of the same framework, small variations in atomic coordinates and/or the treatment of disorder and partial occupancy can move a subset of near-threshold contacts across the bonding criterion.

This can reduce the inferred edge count and increase graph fragmentation, reflected by a higher number of connected components and a lower largest-connected-component fraction. We therefore treat pronounced fragmentation signals as *curation diagnostics* (Class B `TopoOK` flags) that motivate targeted connectivity checks and clean-up, rather than evidence for distinct chemistry or a different underlying net. Full descriptor values for all structures are provided in the released dataset and code repository (GitHub; see Data Availability).
